# Assessment of electronic surveillance and knowledge, attitudes, and practice (KAP) survey toward imported malaria surveillance system acceptance in France

**DOI:** 10.1093/jamiaopen/ooac012

**Published:** 2022-03-09

**Authors:** Marc Thellier, Sandrine Houzé, Bruno Pradine, Renaud Piarroux, Lise Musset, Eric Kendjo, French Imported Malaria Study Group

**Affiliations:** 1 Sorbonne Université, INSERM, Institut Pierre Louis d'Épidémiologie et de Santé Publique, Paris, France; 2 Sorbonne Université, APHP, Hôpital Pitié-Salpêtrière, Service de parasitologie, Paris, France; 3 AP-HP, Centre National de Référence du Paludisme, Paris, France; 4 Parasitology and Mycology Laboratory, Bichat-Claude Bernard Hospital, APHP, Paris, France; 5 Unité Parasitologie et Entomologie, Institut de Recherche Biomédicale des Armées, Institut Hospitalo-Universitaire Méditerranée Infection, Marseille, France; 6 Aix Marseille Université, Marseille, France; 7 IRD, AP-HM, SSA, VITROME, Institut Hospitalo-Universitaire Méditerranée Infection, Marseille, France; 8 Institut Hospitalo-Universitaire Méditerranée Infection, Marseille, France; 9 Laboratoire de Parasitologie, WHO Collaborating Centre for Surveillance of Anti-Malarial Drug Resistance, Centre National de Référence du paludisme, Institut Pasteur de la Guyane, Cayenne, France

**Keywords:** imported malaria, public health, KAP survey, surveillance system, travelers, web-based

## Abstract

**Objective:**

An electronic surveillance system was released to monitor morbidity and mortality incidence of imported malaria cases, investigate autochthonous cases, and assess chemosensitivity of *Plasmodium* isolates among travelers to and from endemic areas. The aim of this study is to evaluate the use of an electronic surveillance system for imported malaria in France.

**Materials and Methods:**

Three main indicators were used to assess the online malaria web-based surveillance system: (1) the quality of the surveillance system; (2) the capacity of the online system to early warning in case of particular events of public health; (3) the knowledge, attitude, and practice of online electronic system by practitioners of malaria network in France.

**Results:**

Overall, the median time onset a case is reported to the system decrease by 99%, ranging from 227 days (144–309) to 2 days (1–6) in 2006 and 2020, respectively.

**Conclusion:**

The online malaria surveillance system in France has demonstrated its effectiveness and can therefore be extended to carry out numerous investigations linked to research on malaria.

## Lay Summary

We describe the surveillance activities of the imported malaria surveillance in travelers from and to endemic areas in France caused by the bite of infected mosquitoes. Furthermore, we evaluate how the participants to the network navigate, appreciate, and report their diagnosed cases to the French National Reference Center for malaria. The main findings are the stability of the network from 1996 through 2020; the reduction of the time between the diagnosis and the declaration of the case in the database. This study provides the effectiveness and ability of this surveillance system to carry out numerous investigations linked to research on malaria and the willingness of their members to participate in the surveillance of imported malaria.

## INTRODUCTION

Malaria surveillance in France is an active surveillance system based on a network integrated into everyday diagnostic procedures conducted by hospital practitioners. Beginning in 1984, imported malaria case reporting moved from paper reports to electronic online notification since 2006. Initially, the Excel spreadsheets have been developed specifically to process the case data from the National Reference Center for Imported and Autochthonous malaria epidemiology collecting epidemiological data (CNREPIA) and the National Reference Center for Malaria Chemosensitivity reporting data on drug resistance (CNRCP). The FNRCm is a secured online system, which collected information on patient infected by *Plasmodium*, including diagnostic, treatment, follow-up, and information for clinician decision-making. Furthermore, the FNRCm provides specialized speciation for all laboratories performing parasitological diagnostics and is a complementary tool for 2 electronic health systems (EHRs) in France (the French national registry on medical causes of death [CepiDc] and the French national hospital discharge database [PMSI]). Therefore, by using this new tool, the FNRCm needed to provide very early warning of malaria conditions in France in order to adjust prevention policies by the public health authorities. However, the EHR is a major challenge within the medicine field, because this activity may combine both low rates of care quality, to higher risk of working inefficiently and experiencing low job satisfaction.^1^ Measurement of usability and acceptance of the technology by physicians is well-described, with several reviews evaluating medical staff barriers and enables to the EHR use and effectiveness.^2^ With regard to the FNRCm, an electronic questionnaire was sent to physicians participating to the network to explore their knowledge, attitudes, and practice (KAP) toward imported malaria surveillance system acceptance in France. The findings of this survey are a part of this evaluation. There is a rich literature on epidemiological, biological, and clinical aspect of malaria in France but, we were unable to find any data assessing or validating the quality of data used for these analyses. The objectives of the present study were to assess the FNRCm online surveillance system and to explore KAP of participants to the network in the case to control and prevent imported malaria in the metropolitan France. To achieve these objectives, the quality of the surveillance system will be assessed, then the capacity of the online system to real-time warning in case of particular events of public health will be evaluated, and finally, we will present the results of the KAP survey.

## MATERIALS AND METHODS

### Data sources

The assessment of the imported malaria surveillance system was done by using data reported by participants to the network. The acceptance of malaria reporting system was based on the KAP survey.

### The KAP survey

In order to maintain the stability and improve the capacity of the FNRCm network during the renewal of the 2012–2016 mandate by the French institute of public health (SPF), a questionnaire was designed to validate the total number of laboratory hospitals that will participate to the surveillance program, including a list of questions to assess the simplicity and acceptability of imported malaria reporting tool. The KAP survey was developed by the members of the FNRCm committee according to the framework for notification. Despite the fact that the usability problem of health information systems is well recognized and that a validated questionnaire to measure usability exists, the questions used in this study were adapted from the daily reporting of imported malaria cases by biologists and clinicians. Three main scores were newly developed, including items on the monitoring tools and functionalities usage; the quality of the online database, and their feedback (Supplementary documents). The questionnaire was tested once in a pilot study by the FNRCm committee members at the national level and the results indicated some changes to the administration methodology and some modifications to be made. However, it is important to notice that the pretest study aimed to adjust the delivery methodology instead of validating of the defined scores. Due to the willingness of biologists and clinicians who are members of the network to report their malaria cases to the FRNCm, all participants in malaria surveillance from 1996 to 2010 received a survey by mail in 2010. An e-mail with the link from questionnaire, login and password were then sent to all participants in the malaria network through the FNRCm platform (Supplementary documents).

### Overview of malaria surveillance in France

Since 1972, the surveillance of infectious and transmissible diseases in France is carried out by SPF, a network of 44 national reference centers (FNRC). The FNRC are public or private health laboratories, generally university hospitals, which diagnose and monitor the incidence of certain diseases. They are appointed for a renewable term of 5 years by the ministry of public health on the proposal of SPF. In France, malaria surveillance system is based on 2 main sources of complementary data: the mandatory reporting of notifiable diseases and the network of clinicians and biologists of the FNRCm. Before 1984, France had no system capable of providing timely surveillance of malaria, therefore, reporting of malaria was mandated by legislation and regulation.^3–5^

### The visibility and dissemination of imported malaria research for policymakers and practitioners

During the 1960s and 1970s, further growth of the FNRCm research was driven largely by biomedical and clinical hospital centers. The primary hypothesis of research on malaria in France regarding the impact of surveillance for public health arises from a considerable literature review concerning cross-sectional observational studies briefly describing the epidemiological characteristics of malaria or presenting clinical cases diagnosed in various public or private hospitals. A first study focused on indigenous malaria cases in France was published in 1954 by the Strasbourg team.^6^^,^^7^ In 1975, Marc Gentilini published a series of 30 malaria cases diagnosed at Pitié-Salpêtrière hospital describing epidemiologic characteristics and management of malaria in Paris.^8^ By following that trend, several other studies on malaria will be published later in different settings and hospitals in France.^9–17^ The first national studies appeared at the end of the 20th century, with the implementation of the FNRCm surveillance system. As a result, the annual number of peer-reviewed articles produced using FNRCm data has increased steadily since 2006. Indeed, from 1996 to 2020, approximately 300 peer-reviewed articles using data from the FNRCm were retrieved from PubMed (Supplementary Figure S1). In addition, the introduction of parenteral artesunate as the first-line treatment for severe malaria in France has highly contributed to the visibility and dissemination of malaria activities in France.^18^^,^^19^ Moreover, the FNRCm is involved at international level in many projects aimed at preventing the introduction and re-emergence of infectious diseases imported into the European Union (EU) and European Economic Area (EEA).^20–25^

### System description

**Figure 1.**
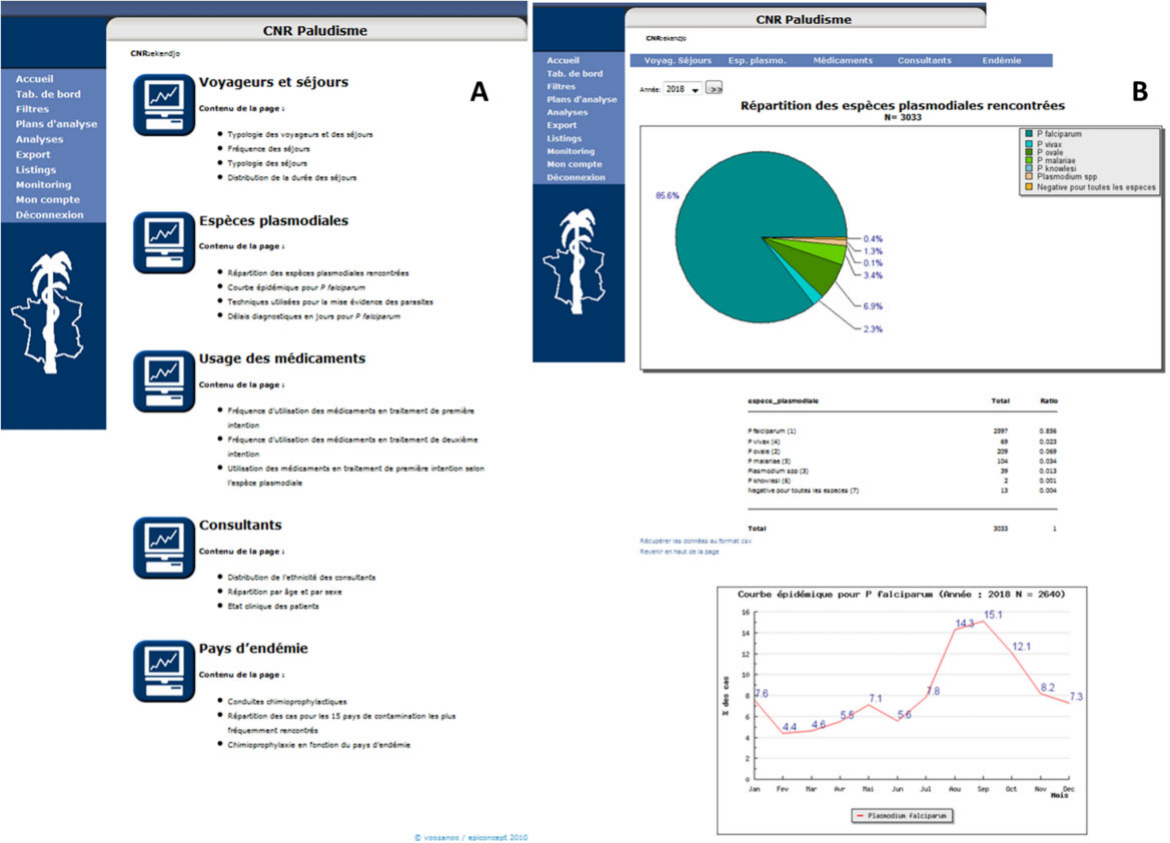
The French national reference surveillance system of malaria: Control panels or dashboards, France, 2006–2018. (A) The menus used for reporting and monitoring malaria cases and blood samples in France. (B) Results of main indicators for malaria surveillance in France.

The current malaria surveillance system is designed around reporting cases diagnosed in France. The FNRCm online surveillance system is based on Linux/Apache/MySQL/PHP framework to customize survey and information system. This platform is licensed under General Public License (GNU/GPL) and benefits of a panel of technologies available for web applications. The questionnaire can be accessed through various internet browsers (ie, *Google chrome, Mozilla, Opera, internet explorer*). The strengths of this tool are the traceability of all actions performed to the database via log files, data storage and processing, archiving, and automated notification of cases (Figure 1). Data providers must maintain the privacy of patients. Therefore, identification codes are automatically generated by the system. Each participant can access to cases under restricted rules. Each malaria case contains a unique key (11-digit alphanumeric code) derived from the year of diagnosis, 3 characters of the hospital name and 4 sequential digits. Data can be easily exported in anonymized flat files (ie, *Excel spreadsheets, text file, PDF*). The communication protocol is encrypted using Transport Layer Security or, formerly, Secure Sockets Layer (ie, *HTTPS protocol*).

### Questionnaire and data collection

Standard case-reporting forms validated by the FNRCm committee members are used to collect malaria cases. Thus, confirmed malaria cases can be registered either to a standard questionnaire with fewer variables or clinical questionnaire with more information for specific research activities on imported malaria (ie, *the surveillance of intravenous artesunate as first-line treatment for severe malaria cases in France*). The questionnaire includes data on demographic details, epidemiology with recent travel history, use of prophylaxis, onset of symptoms, delays in medical care, clinical with initial medical presentation, biology with systematic diagnostic of malaria, and blood cells count.

### Malaria reporting

Participants to the network were asked to report their malaria cases whenever asexual forms of *Plasmodium* were observed from the patient’s blood film directly into the database. On admission, data were collected from patients by physicians under strict confidentiality rules and stored to the database. Some relevant variables were grouped into the sensitive health care category because they may be acquired and used only in conformance with privacy laws or corporate policies (ie, *the date of birth, sex, geographical origin*). These data were needed to create a new malaria case in the database. Data are then reviewed by the FNRCm committee members and all reported cases are investigated further, including induced, congenital, introduced, or cryptic malaria cases. Either way, additional information was requested if needed. More than 60% of hospitals that reporting their cases to the FNRCm also transmitted their blood samples to the FNRCm reference laboratories for diagnostic confirmation, genotyping and phenotyping. Infected patients should be followed-up at Day_3+/−1_, Day_7+/−1_, and Day_28+/−2_ with 2 additional points at Day_14+/−1_ and Day_21+/−1_ as recommended by the health authorities for severe malaria patients treated with intravenous artesunate.

### Ethical statement

The FNRCm surveillance system was approved by the French data protection agency (CNIL) in the declaration number 1223103. Data and samples were all obtained as standard medical care for any patient diagnosed with malaria on hospital admission in the metropolitan France. The collection of blood samples and its components in the context of research activities is regulated by Article L1221-8-1 of the French Public Health Code in France.^26^ According to the French legislation (Article L1211-2 of the French Public Health Code), biomedical database and research resource, containing in-depth genetic and health information can be used several times for scientific purpose as long as informed consent is obtained from patients. In cases involving children, parents or legal representative had to report their opposition to the hospital. According to the French legislation, no institutional review board approval was required in regard of samples from the FNRCm. Since 2016, the FNRCm reference laboratories have been ISO 15189 accredited. They have subscribed to an external quality assessment program for the diagnosis of malaria.

### Data analysis and feedback

Malaria data are annually analyzed for the annual report. The statistical analysis plan is generated by the FNRCm committee members. Data analysis was conducted using the statistical programs JMP^®^pro (Version 15.2.1, SAS Institute Inc., Cary, NC, USA, 1989–2019) and Stata (Version 15.1, StataCorp, College Station, TX, USA). In February, a dataset for previous year is extracted for further analysis. All confirmed malaria cases are controlled for missing data, duplicates, and errors in editing. The data management processes are implemented and scripts are written in both SAS/JMP and Stata programming languages. The public health surveillance systems guideline-based assessment framework from the Center for Disease Control (CDC) was used.^27^ Although some specific indicators are presented here, we do not provide an exhaustive panel. The quality of the surveillance system was evaluated since 1996, before the development of the secured online imported malaria surveillance system. It was characterized firstly, by the stability of hospital providers which is defined as the proportion of cases notified by the corresponding hospitals having at least an identification code, and which constantly reported at least one of their cases to the FNRCm throughout the study period; and then by the completeness of data recorded assessed by using main variables applied to achieve malaria annual report in France. This indicator was defined as a measure of whether all expected data are actually present in a given data set. The capacity of the system was measured using the timeliness of case reporting. Indeed, literature searches have shown that the electronic surveillance improves timeliness and completeness of data.^9^^*,*^^28–32^ To illustrate, presently we would consider data from the FNRCm (eg, *date of reporting, date of diagnosis, number of malaria cases per day reported to the FNRCm, the parasitological control rate*). The actual amount of time required for the FNRCm system to collect or receive data was defined by the onset a case was reported in the database. This indicator plays a leading role in the early warning and response to public health events (ie, *autochthonous cases, emergence of drug resistance*). Furthermore, by using the results from 2 previous studies published by the FNRCm to estimate the sensitivity of the FNRCm for cases and deaths. Finally, an electronic questionnaire was sent to physicians participating to FNRCm network in 2010 to explore their KAP for the reporting surveillance system acceptance. The results of the KAP survey have generated 3 distinct not validated scores for imported malaria cases notification to the FNRCm via secured online electronic surveillance system (ie, *the FNRCm online electronic monitoring system tools and functionalities usage scale, the scale on the quality of the online questionnaire, and the feedback Scale*). For all these scales, the cutoff was defined as the mean of each scale. Therefore, being above the threshold is considered as a positive attitude and perception toward the FNRCm surveillance system. The feedback consists in series of control panel for rapid decision-making and possibility to create data output as table. The annual report is addressed to the SPF in April and validated by the health authorities in June before official dissemination. The data are used for conferences, peer-reviewed articles, and specific communications.

## RESULTS

### The quality of the surveillance system

#### The stability of hospital providers

**Figure 2.**
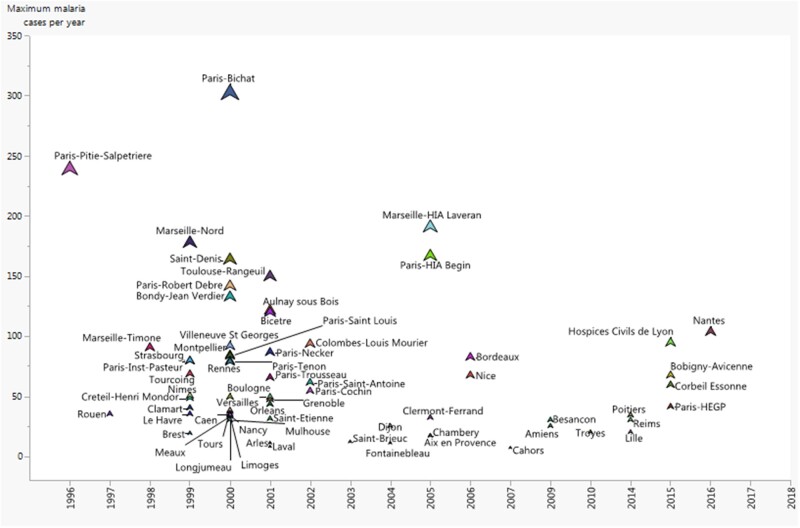
Distribution of the 66 of 172 hospitals that constantly declared their cases to the FNRCm from 17 years up and beyond. *Source:* FNRCm (2019).

**Figure 3.**
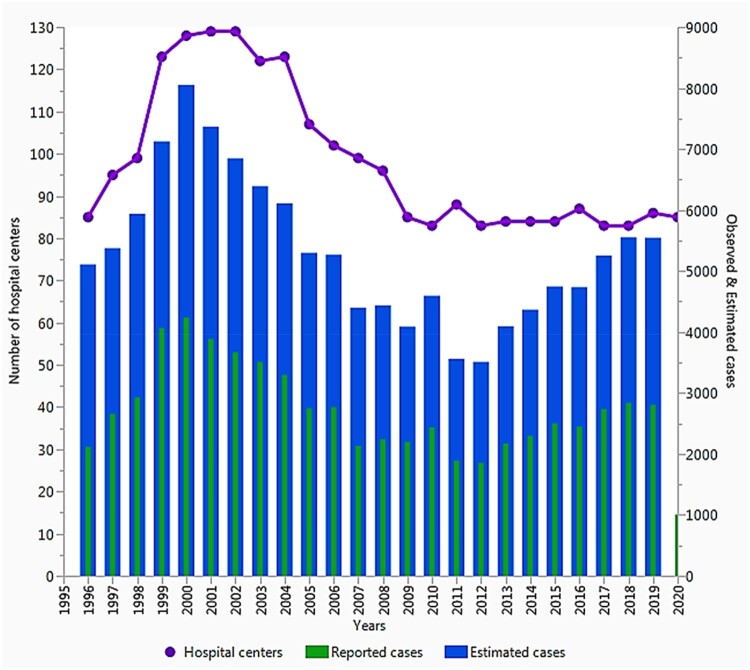
The curve above depicts the distribution of the number of hospitals involved to the FNRCm network; blue bars are the estimated imported malaria cases in travelers returning to the metropolitan France, 1996–2016. The red bars represent cases notified by the corresponding hospitals having at least an identification code, and which constantly reported their cases to the FNRCm throughout the last 2 decades. Purple is the number of hospitals in the French malaria.

From 1996 through 2020, the number of hospital participants to the FNRCm network peaked at 135 hospitals in 2000, then declined and stabilized thereafter to approximately 85 hospitals in 2020, equal to the initial level of 1996 (85 hospitals; Figure 2). But, recent data show an increase in the incidence. Over the last 2 decades, 66 of 172 hospitals that continuously reported their malaria cases to the FNRCm accounted for 85.0% (49 638/58 397) of the total of cases (Figure 3).

#### The completeness of data

The trend in missing data rate for the main variables was summarized in Supplementary Table S1. The results show that the proportion of missing data varied from 0% to 57.4%. Data were exhaustive for the date of diagnosis and *Plasmodium* species. The missing rate decreased for parasitemia, sex, rapid diagnostic test, the purpose of travel, pregnant, immunosuppressed, geographic origin, and the date of birth, while it increased for country of residence, country of birth, clinical type of malaria infection, chemoprophylaxis, date of departure, date of first symptoms, date of returning to France, endemic country visited, first-line treatment, thick smear, and thin smear. Based on the quality of data properly provided without failure, a total of 67 659 malaria cases were reported to the FNRCm from 1996 through 2020. Of these, 29 381 cases were addressed with blood samples from 2006 through 2020 (Figure 2).

### The capacity of the surveillance system

#### Timeliness of case reporting

**Figure 4.**
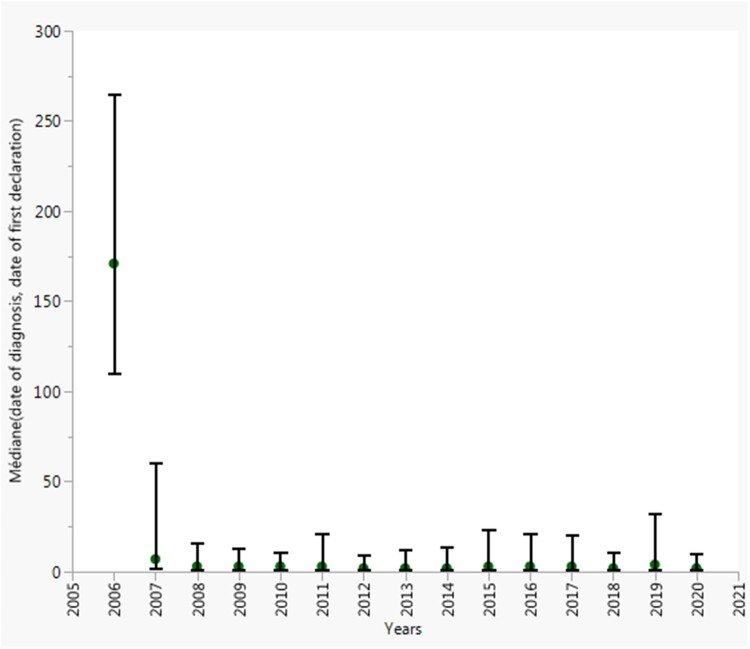
Delay onset cases were reported to the CNR du paludisme database, 2006–2020, France. Points are medians and vertical bars depict the 25e and 75e percentiles.

Across the last 3 decades of malaria surveillance in France, we observed significant decrease in the median onset new cases were reported to the FNRCm, ranged from 227 days (interquartile range [IQR], 144–309) to 2 days (IQR, 1–6) in 2006 and 2020, respectively (Figure 4). Overall, the number of connexion *per day* to the database was 5.5 days (IQR, 0.6–10.5). In addition, the number of cases *per day* reported to the FNRCm was 6.2 days (IQR, 5.1–7.2).

#### The sensitivity of the FNRCm for cases and deaths

The sensitivity of malaria for cases and deaths was defined as the capacity of the network to capture the total number of cases and deaths diagnosed in France.^33–35^ Recent studies have found a large difference in the completeness of malaria-related deaths compares to cases.^36–38^

### The KAP survey

#### Acceptability: the KAP survey

In 2010, a total of 112 participants to the network from 86 hospitals received electronic link to participate to a survey. The response rate was 51.8%. More than half of participants had a higher level of how to use the electronic surveillance system. From those who responded, 98% appreciated reporting their cases to the database and had a positive attitude toward completing their malaria data.

#### The FNRCm online electronic monitoring tools and functionalities usage scale

The FNRCm online electronic monitoring system tools and functionalities usage scale consists of a 10-item survey with dichotomous responses ranging from “higher usage of functionalities and features” to “low use of functionalities and features.” Respondents reported using the FNRCm online electronic tools and functionalities for a mean of 6.2 (range 0–10). Of the 58 respondents for whom these scores were available, 41 (71%) used highly efficient tools and functionalities proposed.

#### The scale on the quality of the online questionnaire

The scale on the quality of the online questionnaire is a 7-item survey with 4-point responses ranging from “higher quality of the online questionnaire” to “low quality of the online questionnaire.” The mean of the score on the quality of the online questionnaire was 11 (range 5–16). Based on the threshold, 57% of the respondents strongly appreciated the quality of the online questionnaire.

#### The feedback scale

The feedback scale is a 10-item survey that summarizes how the main findings from the FNRCm surveillance system are disseminated. A total of 53% of respondents highly appreciated how malaria information and activities are disseminated and vulgarized through workshops, seminars, teaching, and peer-review articles.

## DISCUSSION

**Figure 5.**
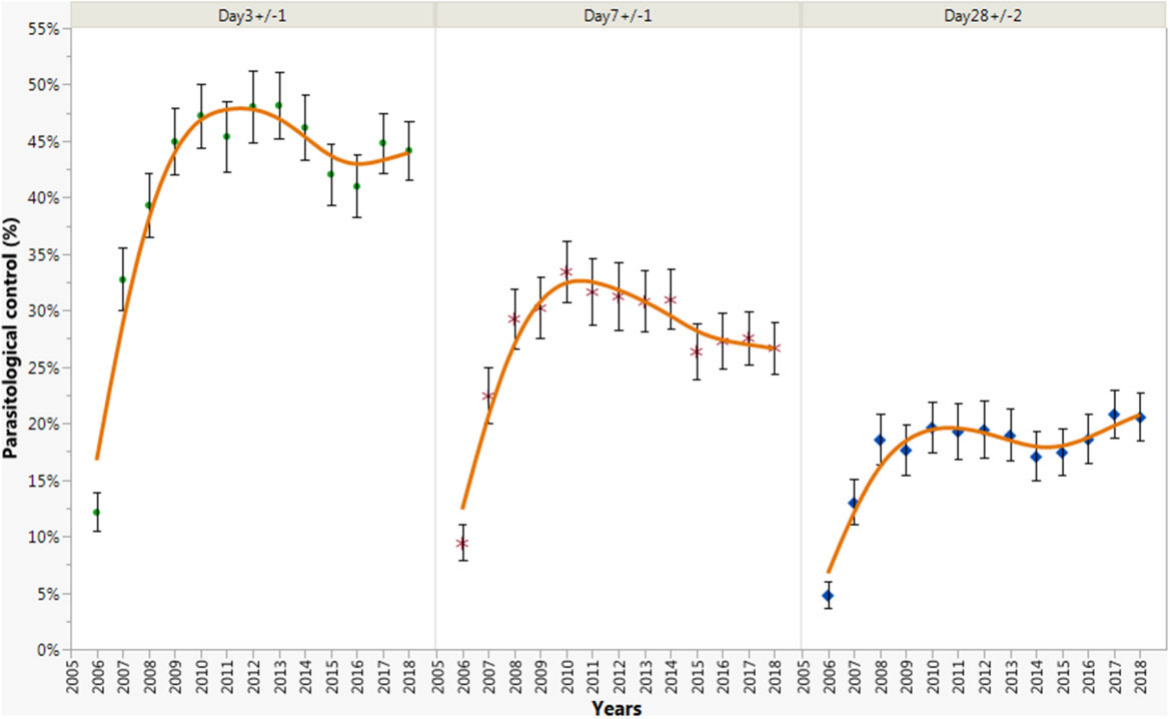
Proportion of parasitological controls at Day_3+/−1_, Day_7+/−1_, and Day_28+/−2_, France, 1996–2018. Points are the proportion of parasitological controls and vertical bars depict the 95% confidence interval. The orange curve represents the kernel density curve.

As well-described in the literature, the FNRCm provides very early warning of malaria health conditions in the metropolitan France based on voluntary participation.^39^ The recent advances using new technology to support imported malaria surveillance program in France have significantly changed the way in which malaria case notification and interaction are occurring through the network. Moreover, there was a significant increase in the proportion of parasitological control from Day_3+/−1_ to Day_28+/__−__2_ from 2006 to 2010, follow by a slightly decrease from 2011 to 2016, except at Day_28+/__−__2_ (Figure 5). At the end of 2020, approximately 67 000 malaria cases, and around 30 000 blood samples have been reported to the FNRCm. Many factors associated with the quality of the surveillance system have been improved. Firstly, the completeness of data collected has increased for most of the main variables used for malaria annual report. This can be explained by the fact that the electronic surveillance systems have huge potential to expand traditional systems, allow several quality control procedures, and increase acceptability.^40^^,^^41^ Milinovich et al^42^ pointed out the fact that internet-based systems are intuitive, adaptable, inexpensive to maintain, and operate in real time. Then, the number of hospitals of the network remains stable during the study period, with 85.0% of cases reported by 66 hospitals of the network. Recently, using data from the FNRCm, Gharbi et al^43^ demonstrated that the FNRCm surveillance system can be used as an additional tool for tracking antimalarial drug resistances in endemic areas. Moreover, beginning in 2011, the FNRCm actually performs the daily monitoring of intravenous artesunate in the treatment of severely infected patients with *Plasmodium falciparum*.^18^^,^^19^^,^^44^

### The sensitivity of the FNRCm for cases and deaths

The pertinent message of this section is the research collaboration established between the FNRCm and the French Public health Agency, SPF for the use of data from the CepiDc and the PMSI in regards to estimating the total number of malaria-related deaths in France. In the same order, we also used data from 3 national exhaustive surveys based on questionnaire sent to all the medical laboratories belonging to the national quality control in parasitology in collaboration with the national agency for the safety of Medical Products (ANSM) to estimate the total incidence of malaria cases in France. Based on this information, France has the UE’s first largest imported malaria incidence,^45^ in which 4 endemic countries (Ivory-Coast, Cameroon, Mali, and the Union of the Comoros) are responsible for 51.2% of all cases.^46–48^ However, progress toward controlling imported malaria incidence had stagnated at around 4000 cases annually since 2007 (Figure 2),^49^ the likelihood of finding a statistically significant reduction was small despite all public health measures to control malaria in France. After additional review and investigation, the FNRCm strategies include the refinement of the web-based surveillance system in a more attractive way for the improvement of responses to emergency situations and epidemics, the promotion and dissemination of malaria activities, and the use of mass campaigns to increase representativeness.^50^ Related to this, the framework used for setting up malaria platform has been upgraded. Therefore, the structure of malaria database has also evolved into patient achieving tools for all imported malaria cases in France (Supplementary Figures S1 and S2). Although the completeness of the FNRCm for cases and deaths in France was comparable to reports elsewhere in the literature, there could be a significant difference in the methods of malaria cases reporting or surveillance system used. Indeed, malaria is a notifiable disease in all EU and EEA countries except Belgium and France.^49^ Taking these insights into a public health research and translational framework on malaria control and vaccine development,^51–56^ under-notification hides the real burden of malaria morbidity and mortality and negatively affects indicators for adequate malaria control and prevention.

### Acceptability: the KAP survey

The most commonly identified barriers to the use of electronic health record were technical problems, perceived redundancy, performance for in-person care, technology anxiety, difficulty remembering to interact with the system, need for technical support, and perceived repetition.^57^ The KAP survey exemplifies the challenges inherent in the use of electronic health record for diseases surveillance, particularly on behavioral attitude and practice in regard of notification. Findings revealed that participants to malaria network have a high level of basic knowledge of how to use the FNRCm web-based surveillance system. They have a positive attitude toward reporting their data to the FNRCm database. The fact that the online surveillance system is functional and practical can justify the strong use of this application. In addition, clinicians and biologists have received initial training or advice from members of the FNRCm or someone from the participating center before reporting their malaria cases to the FNRCm database. In some cases, a short tutorial was sent for beginning. Moreover, after an introduction to navigation, reporting malaria cases seems intuitive. Indeed, several studies have shown that ambulatory physicians frequently express concerns that they spend too much time using the electronic health record^58^^,^^59^ and this tool has been implicated in contributing to physician stress and burnout.^60^ Additionally, the dashboard provides monitoring indicators at the national and regional level.

### Limitations

To make our results more contemporary, we extended some of our analysis until 2020 (see figures and tables). However, the stability of the surveillance system was limited in 2018 due to the fact that Voozanoo application has evolved; therefore, a new framework was generated for imported malaria reporting in France. Moreover, the KAP surveys from 2010, is a bit latter and it was not validated. The nonvalidated survey could yield a significant problem depending on the appropriate score needed which should be standardized for international comparison. These are some focus points to achieve by the FNRCm committee members. We intended to systematically carry out a survey at the end of each year to improve information on participants to malaria network, in addition to validate the stability of the network.

## CONCLUSION

The FNRCm surveillance system has demonstrated its ability to reduce documentation and decrease the time onset data are reported to the database. This review highlighted the commitment, confidence, attitude, and practice of physicians in using the FNRCm secured online monitoring system. Addressing data quality, increasing belief in the use of new technologies, stimulating collaboration, and research funding can foster the will to move forward with the public health missions of the FNRCm.

## AUTHOR CONTRIBUTIONS

EK carried out the initial literature search, contributed to conception and design, participated to the development of FNRCm web-based system, analyzed and interpretation of data, drafted, and edited the manuscript. MT, BP, LM, SH, and RP obtained funding and provided many of the condition definitions, revised the manuscript. All authors read and approved the final manuscript.

## SUPPLEMENTARY MATERIAL

Supplementary material is available at *JAMIA Open* online.

## Supplementary Material

ooac012_Supplementary_Data
