# Assessment of electronic surveillance and knowledge, attitudes, and practice (KAP) survey toward imported malaria surveillance system acceptance in France

**DOI:** 10.1093/jamiaopen/ooac058

**Published:** 2022-07-07

This is a correction to: Marc Thellier, Sandrine Houzé, Bruno Pradine, Renaud Piarroux, Lise Musset, and Eric Kendjo; French Imported Malaria Study Group, Assessment of electronic surveillance and knowledge, attitudes, and practice (KAP) survey toward imported malaria surveillance system acceptance in France, *JAMIA Open*, Volume 5, Issue 1, April 2022, ooac012, https://doi.org/10.1093/jamiaopen/ooac012

In the originally published version of this manuscript the list of “Members of the French Imported Malaria study Group” in the supplementary data was incomplete.

Also the caption for figure 3 incorrectly stated “red bars” when it should have stated “green bars”.

These errors have been corrected.

