# Supplementary material for: Assessment of electronic surveillance and knowledge, attitudes, and practice (KAP) survey toward imported malaria surveillance system acceptance in France
Source: JAMIA Open. 2022 Mar 9;5(1):ooac012. doi: 10.1093/jamiaopen/ooac012 (PMC9097633; doi:10.1093/jamiaopen/ooac012)
Supplement: ooac012_Supplementary_Data [file ooac012_supplementary_data.docx]

## Supplementary material 1. the kap survey


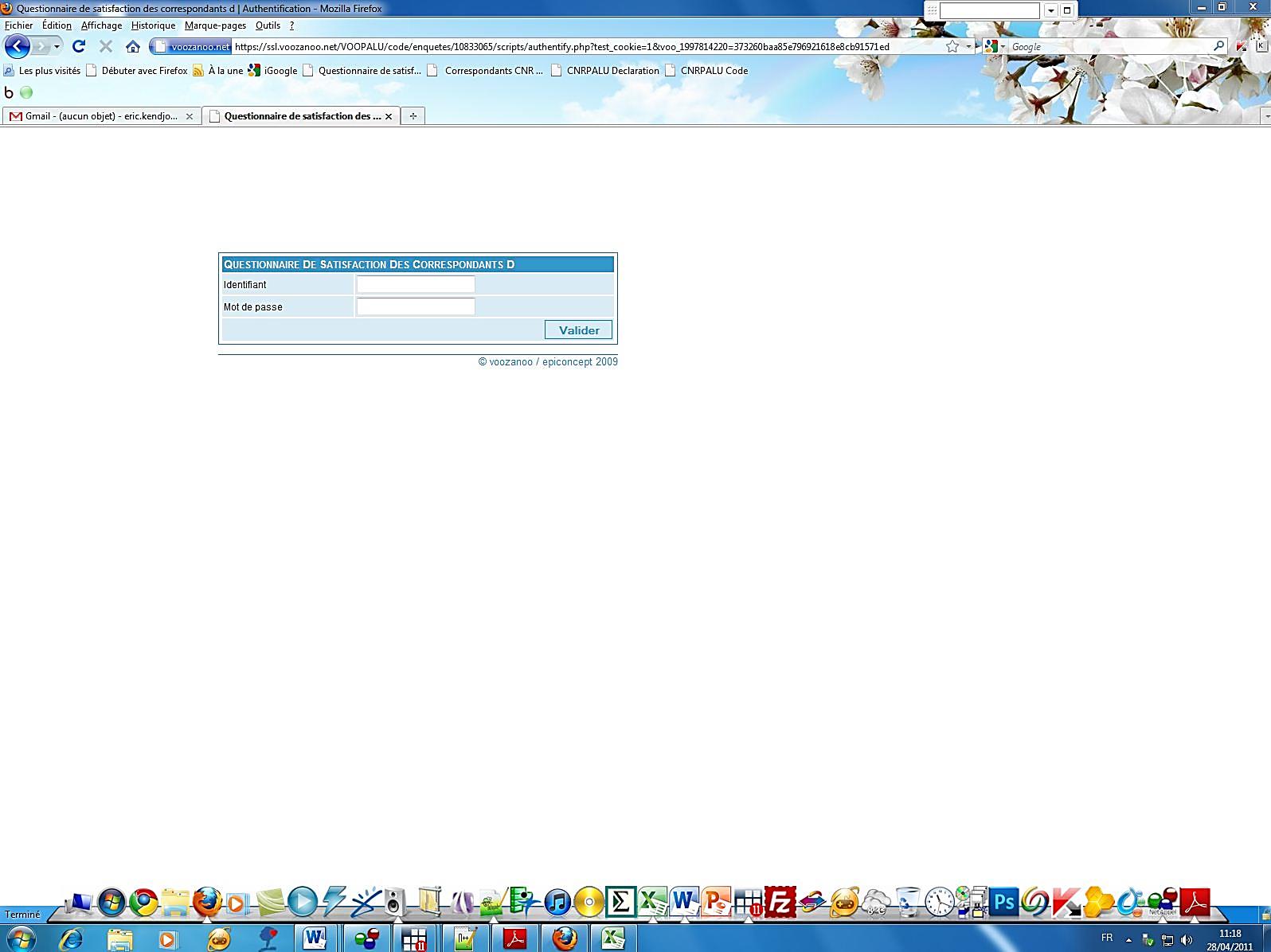


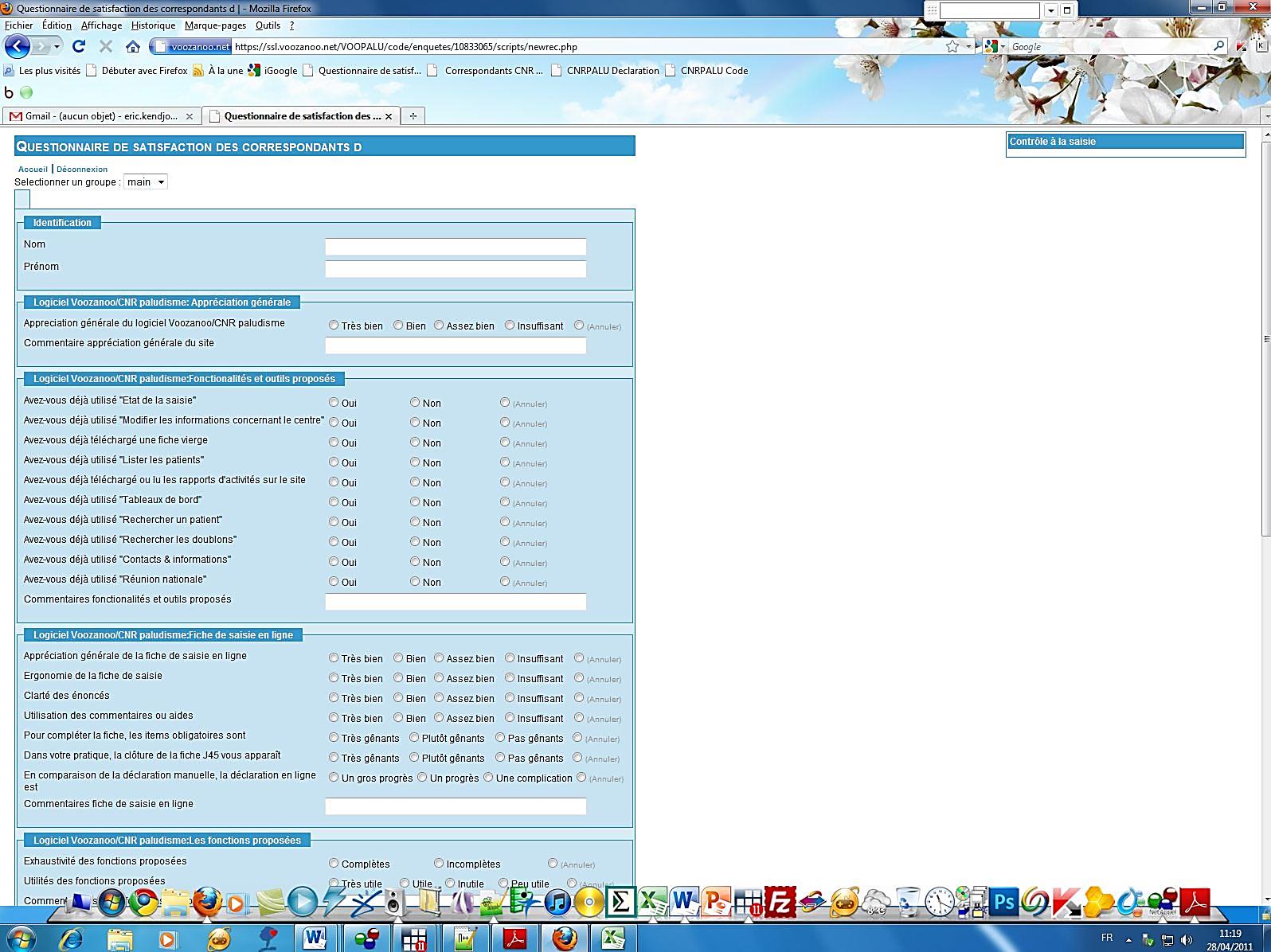


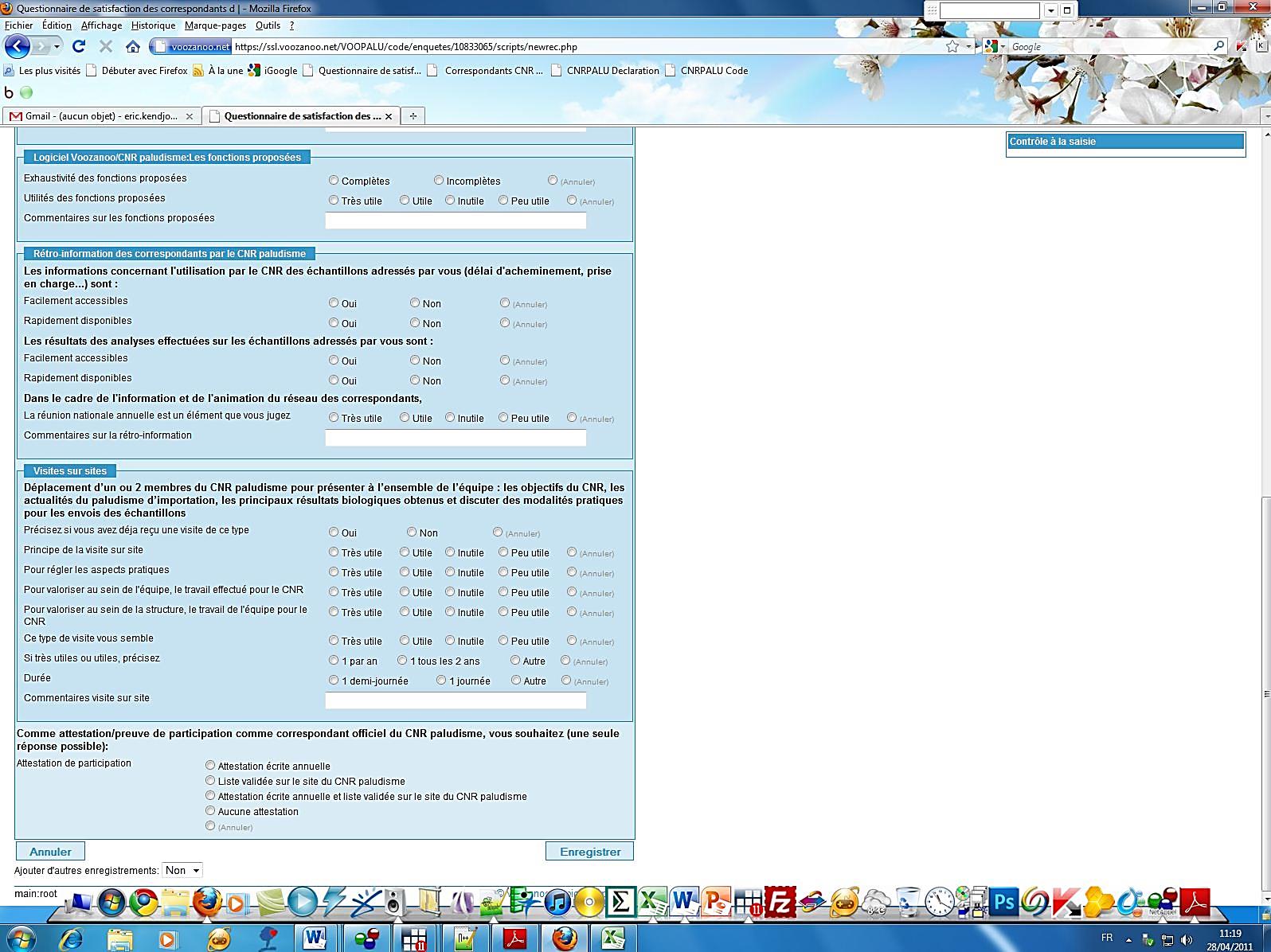


**Supplementary Table 1.***Trend in data missing for the main variables used to editing the malaria annual report of the FNRCm, France, 1996-2016*

|  | 1996 | 1997 | 1998 | 1999 | 2000 | 2001 | 2002 | 2003 | 2004 | 2005 | 2006 | 2007 | 2008 | 2009 | 2010 | 2011 | 2012 | 2013 | 2014 | 2015 | 2016 |
| --- | --- | --- | --- | --- | --- | --- | --- | --- | --- | --- | --- | --- | --- | --- | --- | --- | --- | --- | --- | --- | --- |
| Chemoprolaxis | 9.58% | 11.25% | 8.07% | 9.32% | 9.52% | 8.68% | 11.02% | 9.55% | 9.37% | 8.46% | 6.54% | 6.63% | 5.17% | 6.18% | 7.50% | 8.10% | 6.79% | 10.80% | 9.56% | 15.04% | 14.75% |
| Clinical history |  |  |  |  |  |  |  | 7.49% | 15.94% | 27.47% | 23.49% | 20.65% | 17.15% | 19.93% | 24.56% | 27.66% | 30.68% | 37.88% | 35.01% | 38.29% | 37.35% |
| Clinical type of malaria | 0.38% | 1.02% | 0.38% | 4.13% | 7.07% | 7.18% | 0.90% | 0.57% | 0.00% | 2.01% | 4.03% | 2.63% | 1.92% | 1.29% | 0.45% | 0.65% | 0.58% | 2.76% | 1.25% | 5.05% | 5.21% |
| Country of birth | 13.47% | 11.93% | 3.66% | 0.47% | 11.13% | 11.08% | 15.63% | 17.87% | 26.39% | 14.09% | 22.33% | 17.48% | 15.95% | 13.12% | 12.93% | 17.51% | 13.06% | 21.34% | 17.96% | 28.35% | 30.71% |
| Country of residence | 13.37% | 3.95% | 0.75% | 0.54% | 0.73% | 7.10% | 1.15% | 1.52% | 2.12% | 6.30% | 12.27% | 9.26% | 8.06% | 6.98% | 5.07% | 7.90% | 2.49% | 17.04% | 5.94% | 11.25% | 19.23% |
| Date of birth | 3.27% | 2.41% | 1.33% | 1.67% | 1.02% | 1.27% | 0.82% | 1.15% | 1.23% | 1.98% | 0.00% | 0.05% | 0.00% | 0.00% | 0.04% | 0.00% | 0.00% | 0.00% | 0.00% | 0.00% | 0.00% |
| Date of departure | 35.04% | 32.36% | 32.00% | 32.60% | 46.03% | 41.21% | 45.38% | 42.97% | 35.88% | 38.73% | 34.28% | 36.54% | 37.55% | 39.06% | 42.40% | 47.97% | 44.64% | 48.33% | 43.07% | 46.98% | 52.26% |
| Date of first symptoms | 2.94% | 6.25% | 2.67% | 7.30% | 1.72% | 15.93% | 19.42% | 22.72% | 15.12% | 16.85% | 14.02% | 12.03% | 12.34% | 9.16% | 10.82% | 14.86% | 9.24% | 18.63% | 16.54% | 18.56% | 21.59% |
| Date of return to France | 5.17% | 4.93% | 1.91% | 6.17% | 4.68% | 12.70% | 4.01% | 4.30% | 5.41% | 14.83% | 4.58% | 8.62% | 7.26% | 9.16% | 10.42% | 12.11% | 10.30% | 10.99% | 7.84% | 11.37% | 12.38% |
| Endemic country | 0.00% | 0.08% | 0.17% | 0.07% | 0.02% | 0.00% | 0.03% | 0.00% | 0.12% | 0.56% | 0.00% | 0.05% | 0.09% | 0.00% | 0.00% | 0.00% | 0.32% | 1.49% | 1.03% | 3.59% | 4.73% |
| First-line treatment | 6.21% | 4.59% | 3.15% | 6.19% | 5.17% | 5.99% | 6.24% | 6.74% | 5.44% | 6.08% | 6.35% | 5.63% | 5.43% | 5.16% | 6.12% | 5.85% | 3.61% | 7.55% | 5.68% | 10.46% | 14.83% |
| Geographic origin | 13.37% | 11.66% | 3.62% | 0.37% | 10.82% | 4.13% | 14.73% | 12.11% | 12.29% | 11.93% | 0.00% | 0.05% | 0.13% | 0.00% | 0.00% | 0.10% | 0.42% | 5.88% | 3.75% | 7.82% | 10.26% |
| Immunosuppressed |  |  |  |  |  |  |  |  |  |  | 37.62% | 19.25% | 18.35% | 18.42% | 17.19% | 21.36% | 19.43% | 21.84% | 16.71% | 16.46% | 15.80% |
| Malaria symptoms |  |  |  |  |  |  |  |  |  |  |  |  |  |  |  |  |  | 16.00% | 6.42% | 8.65% | 10.47% |
| Pregnant | 23.94% | 23.89% | 23.79% | 24.58% | 25.64% | 26.36% | 25.80% | 24.04% | 22.70% | 23.74% | 6.72% | 3.99% | 3.88% | 4.36% | 5.43% | 5.00% | 4.51% | 5.38% | 3.62% | 4.26% | 3.34% |
| Purpose of travel | 57.42% | 55.15% | 42.15% | 36.58% | 20.01% | 15.31% | 14.10% | 9.24% | 9.31% | 9.69% | 9.26% | 9.90% | 10.16% | 8.14% | 9.36% | 10.56% | 6.48% | 22.20% | 18.17% | 24.44% | 30.26% |
| RDT |  |  |  |  |  |  |  |  |  |  |  |  |  |  |  |  |  | 13.38% | 5.04% | 4.22% | 3.95% |
| Sex | 0.28% | 0.19% | 0.21% | 0.05% | 0.02% | 0.00% | 0.16% | 0.11% | 0.00% | 0.17% | 0.15% | 0.09% | 0.09% | 0.13% | 0.00% | 0.00% | 0.05% | 0.05% | 0.00% | 0.00% | 0.00% |
| Thick smear |  |  |  |  |  |  |  |  |  |  | 51.42% | 25.10% | 25.52% | 24.11% | 21.48% | 22.16% | 15.61% | 14.01% | 12.49% | 11.29% | 13.32% |
| Thin smear |  |  |  |  |  |  |  |  |  |  | 2.65% | 1.77% | 1.60% | 2.71% | 0.77% | 2.15% | 1.96% | 2.17% | 1.29% | 1.70% | 1.96% |
| Parasitemia | 24.32% | 19.94% | 17.95% | 22.22% | 16.75% | 16.73% | 17.04% | 23.03% | 18.80% | 20.57% | 21.82% | 19.56% | 18.75% | 17.93% | 14.35% | 16.01% | 9.45% | 13.65% | 12.23% | 10.98% | 11.98% |

** All empty cells characterized the absence of these variables in that period*

**
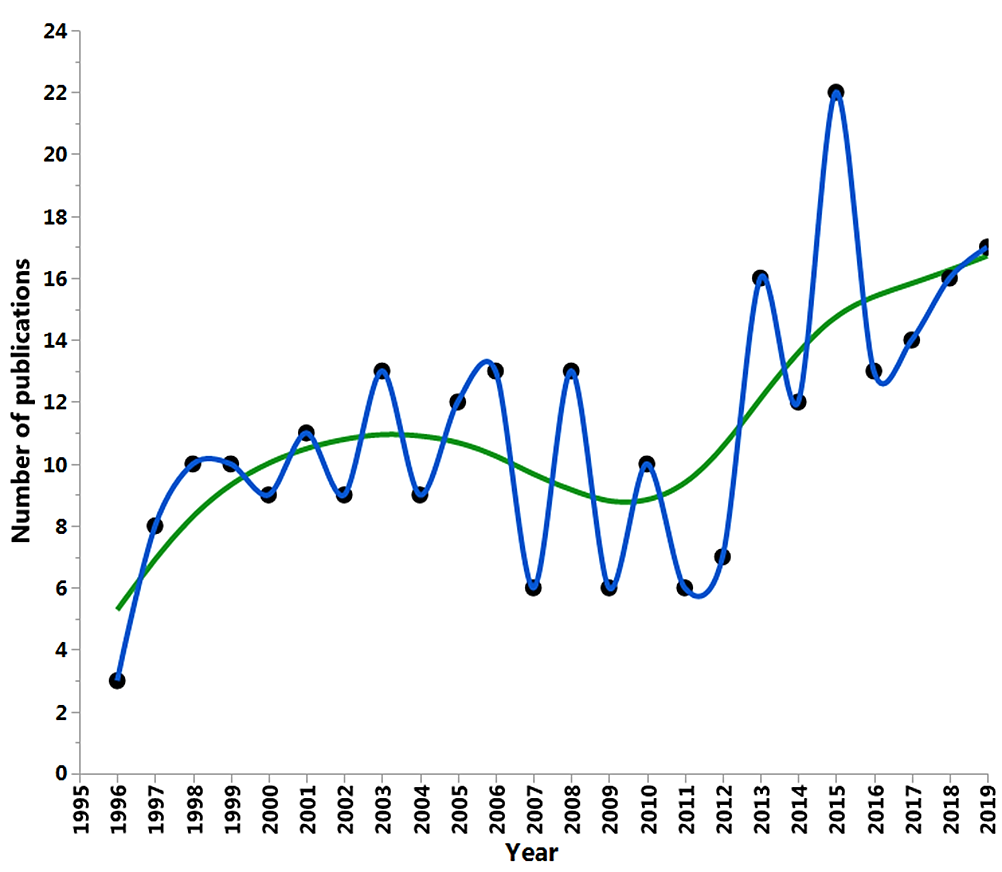
**

**Supplementary figure 1.** The annual number of peer-reviewed papers from the FNRCm, France, 1996-2019. Points are the median number of peer-reviewed papers and the green curve represents the kernel density curve.

**
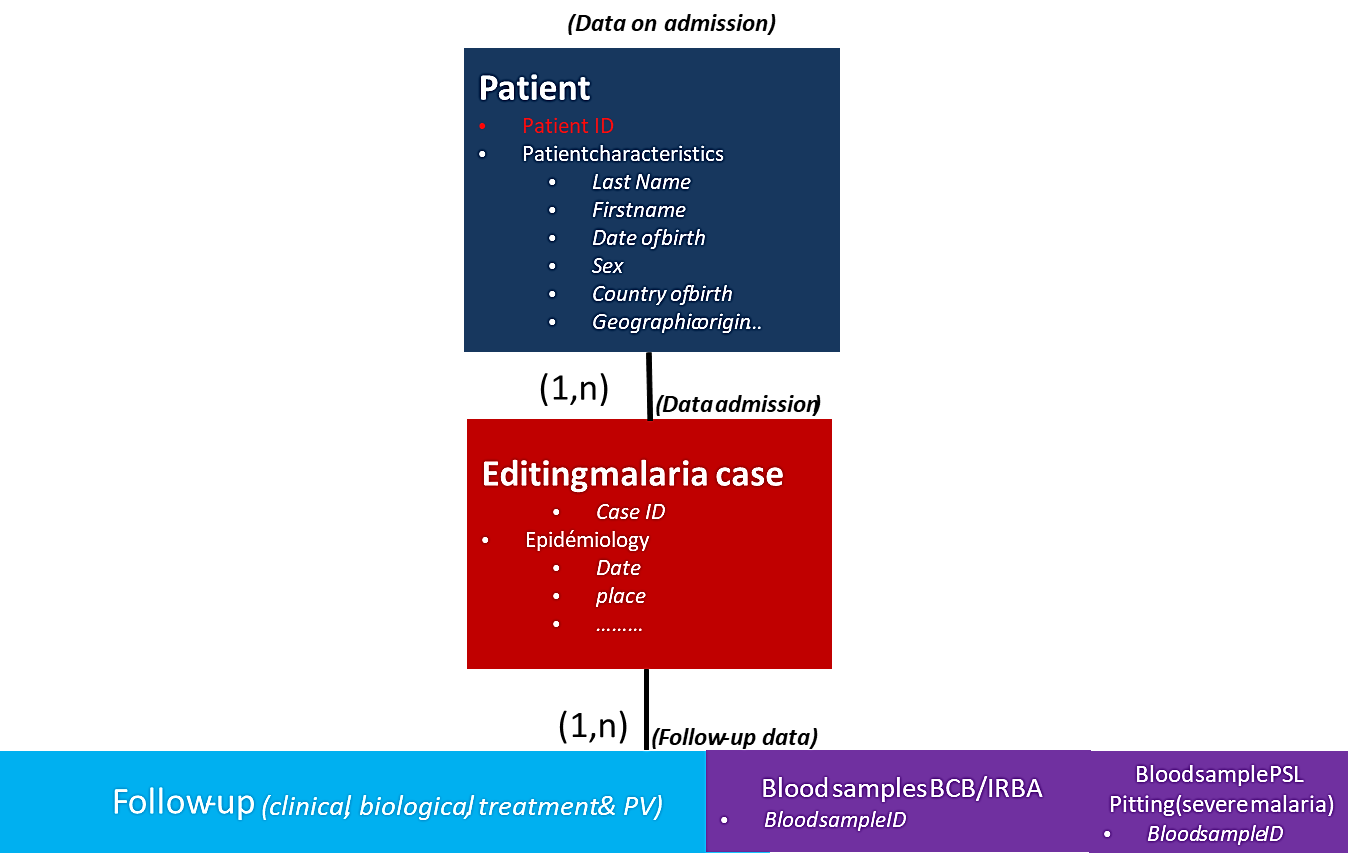
**

**Supplementary figure 2.** The structure of the FNRCm database

**
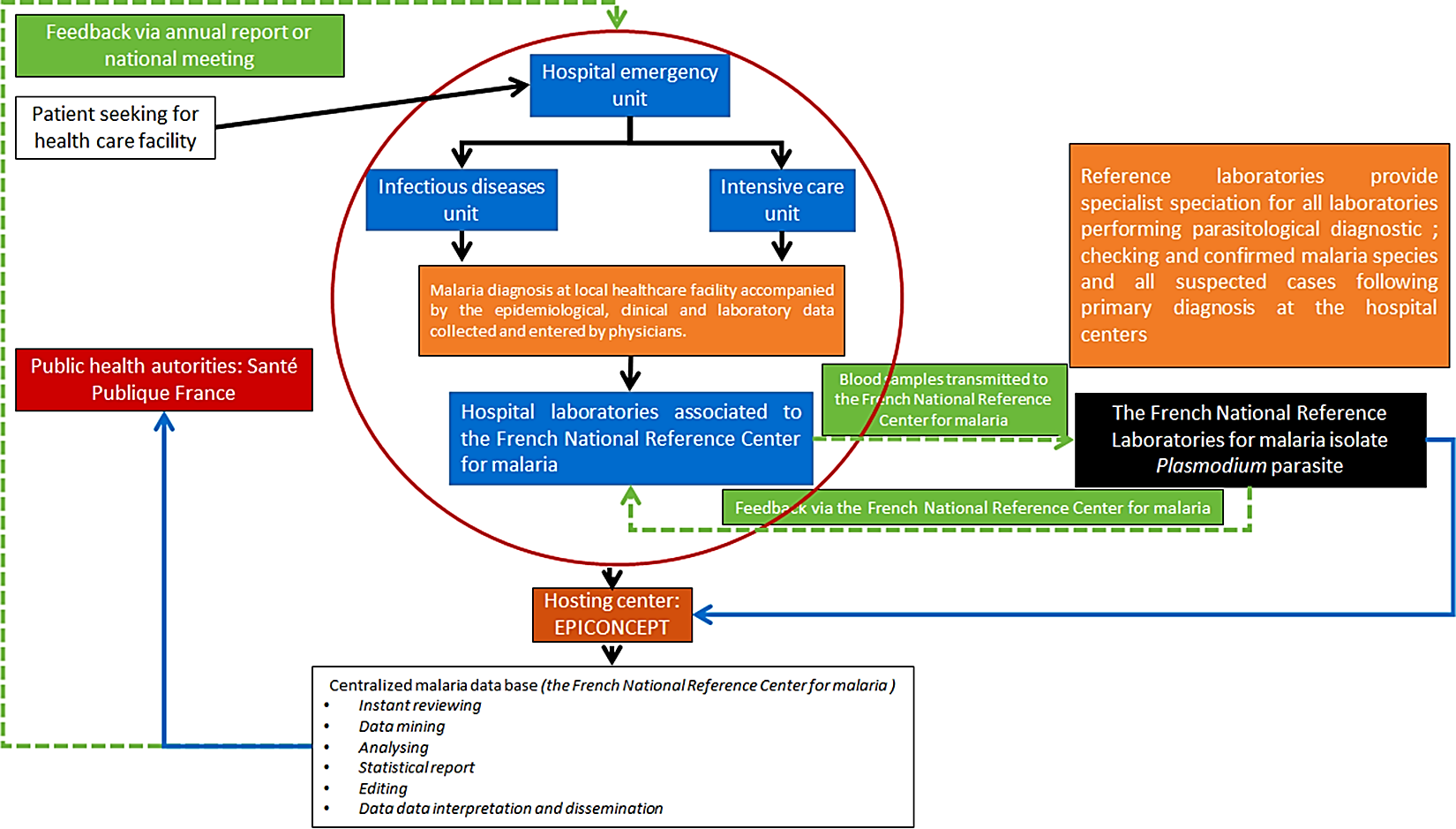
**

**Supplementary figure 3**. Internet-based reporting system working principle of the French imported malaria network, France, 2006-2016. Data can be collected both at the hospital, in a reference laboratory, or directly by the FNRCm members.

**Members of the French Imported Malaria study Group are:**

| **CITY** | **TITLE** | **NAME** | **HOSPITAL** | **DEPARTMENT** |
| --- | --- | --- | --- | --- |
| AIX EN PROVENCE | DR | ADRIEN GENIN | CH PAYS D'AIX | LABORATOIRE HEMATOLOGIE-IMMUNOLOGIE |
| AIX EN PROVENCE | DR | ALAIN DOMERGUES | CH PAYS D'AIX | LABORATOIRE HEMATOLOGIE-IMMUNOLOGIE |
| AIX EN PROVENCE | DR | CHANTAL GARABEDIAN | CH PAYS D'AIX | LABORATOIRE HEMATOLOGIE-IMMUNOLOGIE |
| ALBI | DR | MONIQUE GREZE | CH ALBI | LABORATOIRE DE BIOLOGIE MEDICALE |
| ALES | DR | LUCILE CADOT | CH ALES CEVENNES | LABORATOIRE DE BIOLOGIE MEDICALE |
| AMIENS | DR | ALICE BOREL | CHU AMIENS-SUD | SERVICE DES MALADIES INFECTIEUSES |
| AMIENS | DR | CELINE DAMIANI | CHU AMIENS-SUD | LABORATOIRE DE PARASITOLOGIE-MYCOLOGIE |
| AMIENS | DR | PATRICE AGNAMEY | CHU AMIENS-SUD | LABORATOIRE DE PARASITOLOGIE - MYCOLOGIE |
| AMIENS | DR | Yohann LE GOVIC | CHU AMIENS-SUD | LABORATOIRE DE PARASITOLOGIE-MYCOLOGIE |
| ANGERS | DR | JEAN-PHILIPPE LEMOINE | CHU ANGERS | LABORATOIRE DE PARASITOLOGIE-MYCOLOGIE |
| ANGERS | DR | LUDOVIC DE GENTILE | CHU ANGERS | LABORATOIRE DE PARASITOLOGIE-MYCOLOGIE |
| ANGERS | DR | MARC PIHET | CHU ANGERS | LABORATOIRE DE PARASITOLOGIE-MYCOLOGIE |
| ANGERS | DR | VINCENT DUBEE | CHU ANGERS | LABORATOIRE DE PARASITOLOGIE-MYCOLOGIE |
| ANGERS | DR | YOHANN LE GOVIC | CHU ANGERS | LABORATOIRE DE PARASITOLOGIE-MYCOLOGIE |
| ANGOULEME | DR | CAROLINE GARANDEAU | CH ANGOULEME | LABORATOIRE DE PARASITOLOGIE-MYCOLOGIE |
| ANNECY | DR | MACHEDA GABRIEL | CH ANNECY | SERVICE DES MALADIES INFECTIEUSES |
| ANNECY | DR | PIERRE MORNAND | CH ANNECY | SERVICE DE PEDIATRIE |
| ARLES | DR | MURIEL ROUMIER | CH ARLES | LABORATOIRE DE BIOLOGIE MEDICALE |
| AULNAY-SOUS-BOIS | DR | ANNE DELAVAL | CHIR BALLANGER | LABORATOIRE DE PARASITOLOGIE-MYCOLOGIE |
| AULNAY-SOUS-BOIS | DR | CECILE LEPRINCE | CHIR BALLANGER | LABORATOIRE DE PARASITOLOGIE-MYCOLOGIE |
| AULNAY-SOUS-BOIS | DR | ELODIE COLLIN | CHIR BALLANGER | LABORATOIRE DE PARASITOLOGIE-MYCOLOGIE |
| AUXERRE | DR | NAYLA NASSAR | CH AUXERRE | LABORATOIRE DE PARASITOLOGIE-MYCOLOGIE |
| AVICENNE/BONDY | DR | ANTHONY MARTEAU | CH AVICENNE | LABORATOIRE DE PARASITOLOGIE-MYCOLOGIE |
| AVICENNE/BONDY | DR | AREZKI IZRI | CH AVICENNE | LABORATOIRE DE PARASITOLOGIE-MYCOLOGIE |
| BESANCON | DR | ANNE PAULINE BELLANGER | CHU JEAN MINJOZ | LABORATOIRE DE PARASITOLOGIE-MYCOLOGIE |
| BESANCON | DR | EMELINE SCHERER | CHU JEAN MINJOZ | LABORATOIRE DE PARASITOLOGIE-MYCOLOGIE |
| BESANCON | PR | LAURENCE MILLON | CHU JEAN MINJOZ | LABORATOIRE DE PARASITOLOGIE-MYCOLOGIE |
| BESANCON | DR | TIMOTHEE KLOPFENSTEIN | CHU JEAN MINJOZ | SERVICE DES MALADIES INFECTIEUSES |
| BEZIERS | DR | MARION ALMERAS | CH BEZIERS | LABORATOIRE DE PARASITOLOGIE-MYCOLOGIE |
| BEZIERS | DR | ODILE FALGUIERE | CH BEZIERS | LABORATOIRE DE PARASITOLOGIE-MYCOLOGIE |
| BIOLOGISTE DU CNR | DR | BAILLY | CNR - BIOLOGISTE DU CNR |  |
| BIOLOGISTE DU CNR | DR | CARLA DA CUNHA | CNR - BIOLOGISTE DU CNR |  |
| BIOLOGISTE DU CNR | DR | RIZWANA ZAFFAROULLAH | CNR - BIOLOGISTE DU CNR |  |
| BIOLOGISTE DU CNR | DR | THOMAS CORBETT | CNR - BIOLOGISTE DU CNR |  |
| BIOLOGISTE DU CNR | DR | VALENTIN JOSTE | CNR - BIOLOGISTE DU CNR |  |
| BLOIS | DR | CECILE HOMBROUCK-ALET | CH BLOIS | LABORATOIRE DE PARASITOLOGIE-MYCOLOGIE |
| BLOIS | DR | LAURENT GUILLAUME | CH BLOIS | LABORATOIRE DE PARASITOLOGIE-MYCOLOGIE |
| BOBIGNY | DR | OLIVIER BOUCHAUD | CH AVICENNE | SERVICE DES MALADIES INFECTIEUSES |
| BORDEAUX | PR | DENIS MALVY | CHU BORDEAUX | SERVICE DE MALADIES INFECTIEUSES |
| BORDEAUX | DR | MARIE-CATHERINE RECEVEUR | CHU BORDEAUX | UNITES MALADIES TROPICALES ET DU VOYAGEUR |
| BORDEAUX | DR | MATTHIEU MECHAIN | CHU BORDEAUX | UNITES MALADIES TROPICALES ET DU VOYAGEUR |
| BORDEAUX | DR | STEPHANIE DULUCQ | CHU BORDEAUX | LABORATOIRE DE PARASITOLOGIE-MYCOLOGIE |
| BORDEAUX | DR | THIERRY PISTONE | CHU BORDEAUX | UNITES MALADIES TROPICALES ET DU VOYAGEUR |
| BORDEAUX | DR | VALERIE FUSTER-DUMAS | CHU BORDEAUX | LABORATOIRE D'HEMATOLOGIE |
| BOULOGNE | DR | JEAN DUNAND | CH BOULOGNE | LABORATOIRE DE PARASITOLOGIE-MYCOLOGIE |
| BOULOGNE | DR | SYLVAIN CLAUSER | CH BOULOGNE | LABORATOIRE DE PARASITOLOGIE-MYCOLOGIE |
| BOULOGNE | PR | THOMAS HANSLIK | CH BOULOGNE | LABORATOIRE DE PARASITOLOGIE-MYCOLOGIE |
| BOURGES | DR | AURORE SANSON | CH JACQUES CŒUR | LABORATOIRE DE BIOLOGIE MEDICALE |
| BOURGES | DR | CARINE DOKOULA | CH JACQUES CŒUR | MEDECINE INTERNE - MALADIES INFECTIEUSES |
| BOURGES | DR | GUY CARROGER | CH JACQUES CŒUR | LABORATOIRE DE BIOLOGIE MEDICALE |
| BOURGES | DR | MARIE-NADEGE BACHELIER | CH JACQUES CŒUR | LABORATOIRE DE BIOLOGIE MEDICALE |
| BOURGES | DR | YVES GUIMARD | CH JACQUES CŒUR | MEDECINE INTERNE - MALADIES INFECTIEUSES |
| BREST | DR | DOROTHEE QUINIO | CHU CAVALE BLANCHE | LABORATOIRE DE PARASITOLOGIE-MYCOLOGIE |
| BREST | PR | GILLES NEVEZ | CHU CAVALE BLANCHE | LABORATOIRE DE PARASITOLOGIE-MYCOLOGIE |
| BREST | DR | CATHERINE DESIDERI-VAILLANT | HIA BREST-CLERMONT-TONNERRE | LABORATOIRE DE BIOLOGIE MEDICALE |
| BREST | DR | JEAN-LUC VIGNOT | HIA BREST-CLERMONT-TONNERRE | LABORATOIRE DE BIOLOGIE MEDICALE |
| BREST | DR | JEANNE SAPIN-LORY | HIA BREST-CLERMONT-TONNERRE | LABORATOIRE DE BIOLOGIE MEDICALE |
| BREST | DR | LAURENCE POUGNET | HIA BREST-CLERMONT-TONNERRE | LABORATOIRE DE PARASITOLOGIE - MYCOLOGIE |
| BREST | DR | SIMON RAMBAUD | HIA BREST-CLERMONT-TONNERRE | LABORATOIRE DE BIOLOGIE MEDICALE |
| BRETIGNY-SUR-ORGE | DR | MYLENE PENOT | CERBA/IMTSSA | UNITE DE TOXICOLOGIE ANALYTIQUE |
| BRETIGNY-SUR-ORGE | DR | NICOLAS TAUDON | CERBA/IMTSSA | UNITE DE TOXICOLOGIE ANALYTIQUE |
| CAEN | DR | AGATHE CAPITAINE | CHU COTE DE NACRE | LABORATOIRE DE MICROBIOLOGIE |
| CAEN | DR | JULIE BONHOMME | CHU COTE DE NACRE | LABORATOIRE DE MICROBIOLOGIE |
| CAHORS | DR | NATHALIE WILHELM | CH JEAN ROUGIER | SERVICE DE BIOLOGIE MEDICALE ET PHYSIOLOGIE |
| CANNES | DR | ISABELLE SALIMBENI | CH CANNES | LABORATOIRE DE BIOLOGIE MEDICALE |
| CAYENNE | DR | FELIX DJOSSOU | CH CAYENNE ANDREE ROSEMON | SERVICE D'INFECTIOLOGIE (UMIT) |
| CAYENNE | DR | STEPHANE PELLEAU | CH CAYENNE ANDREE ROSEMON | LABORATOIRE DE PARASITOLOGIE - MYCOLOGIE |
| CAYENNE | DR | CHRISTELLE PRINCE | INSTITUT PASTEUR DE GUYANE | LABORATOIRE DE PARASITOLOGIE - MYCOLOGIE |
| CAYENNE | DR | CLAUDE FLAMAND | INSTITUT PASTEUR DE GUYANE | LABORATOIRE DE BIOLOGIE MEDICALE |
| CAYENNE | DR | LISE MUSSET | INSTITUT PASTEUR DE GUYANE | LABORATOIRE DE PARASITOLOGIE - MYCOLOGIE |
| CAYENNE | DR | YASSAMINE LAZREK | INSTITUT PASTEUR DE GUYANE | LABORATOIRE DE PARASITOLOGIE - MYCOLOGIE |
| CHAMBERY | DR | DIDIER RAFFENOT | CH CHAMBERY | LABORATOIRE DE PARASITOLOGIE - MYCOLOGIE |
| CHAMBERY | DR | OLIVIER ROGEAUX | CH CHAMBERY | SERVICE DES MALADIES INFECTIEUSES |
| CHERBOURG EN COTENTIN | DR | G. MARTIN | CH CHERBOURG | LABORATOIRE DE PARASITOLOGIE-MYCOLOGIE |
| CHERBOURG EN COTENTIN | DR | JOSETTE JEHAN | CH CHERBOURG | LABORATOIRE DE PARASITOLOGIE-MYCOLOGIE |
| CHOLET | DR | PAULINE TOUROULTJUPIN | CH CHOLET | LABORATOIRE DE PARASITOLOGIE - MYCOLOGIE |
| CHOLET | DR | ROXANE COURTOIS | CH CHOLET | LABORATOIRE DE PARASITOLOGIE - MYCOLOGIE |
| CHOLET | DR | TIPHAINE MERGEY | CH CHOLET | LABORATOIRE DE PARASITOLOGIE - MYCOLOGIE |
| CLAMART | DR | ANNE MARFAING-KOKA | CH CLAMART | LABORATOIRE DE PARASITOLOGIE - MYCOLOGIE |
| CLAMART | DR | COURNAC JEAN-MARIE | CH CLAMART | LABORATOIRE DE PARASITOLOGIE - MYCOLOGIE |
| CLERMONT-FERRAND | DR | NOURRISSON CELINE | CHRU CLERMONT-FERRAND | LABORATOIRE DE PARASITOLOGIE-MYCOLOGIE |
| CLERMONT-FERRAND | DR | PHILIPPE POIRIER | CHRU CLERMONT-FERRAND | LABORATOIRE DE PARASITOLOGIE-MYCOLOGIE |
| COLOMBES-LOUIS MOURIER | DR | AHMED FATEH OUSSER | CH LOUIS-MOURIER | SERVICE DE MICROBIOLOGIE ET HYGIENE |
| COLOMBES-LOUIS MOURIER | DR | ANNE-CLAIRE MAHERAULT | CH LOUIS-MOURIER | SERVICE DE MICROBIOLOGIE ET HYGIENE |
| COLOMBES-LOUIS MOURIER | DR | CARMINA CAMAL | CH LOUIS-MOURIER | SERVICE DE MICROBIOLOGIE ET HYGIENE |
| COLOMBES-LOUIS MOURIER | DR | LUCE LANDRAUD | CH LOUIS-MOURIER | SERVICE DE MICROBIOLOGIE ET HYGIENE |
| COLOMBES-LOUIS MOURIER | DR | MARTINE BLOCH | CH LOUIS-MOURIER | MEDECINE INTERNE |
| COLOMBES-LOUIS MOURIER | DR | NATHALIE PAREZ | CH LOUIS-MOURIER | SERVICE DE MICROBIOLOGIE ET HYGIENE |
| COLOMBES-LOUIS MOURIER | DR | RAHAF HAJ HAMID | CH LOUIS-MOURIER | SERVICE DE MICROBIOLOGIE ET HYGIENE |
| CORBEIL ESSONNE | DR | SABAH KUBAB | CH CORBEIL ESSONNE | LABORATOIRE DE BIOLOGIE MEDICALE |
| COTENTIN | DR | JAN JEHAN | CH COTENTIN | LABORATOIRE DE PARASITOLOGIE-MYCOLOGIE |
| CREIL | DR | ANGELE LI | GHPSO | LABORATOIRE DE BIOLOGIE MEDICALE |
| CREIL | DR | CHRISTINE CHAIGNEAU | GHPSO | LABORATOIRE DE BIOLOGIE MEDICALE |
| CRETEIL | DR | FRANÇOISE BOTTEREL | CH CRETEIL-HENRI MONDOR | LABORATOIRE DE PARASITOLOGIE-MYCOLOGIE |
| CRETEIL | DR | NATHALIE FAUCHET | CH CRETEIL-HENRI MONDOR | LABORATOIRE DE PARASITOLOGIE-MYCOLOGIE |
| CRETEIL | DR | REMY DURAND | CH CRETEIL-HENRI MONDOR | LABORATOIRE DE PARASITOLOGIE - MYCOLOGIE |
| DIJON | DR | BASMACIYAN LOUISE | CH DIJON | LABORATOIRE DE PARASITOLOGIE-MYCOLOGIE |
| DOURDAN | DR | C. FARRUGIA | CH DOURDAN | LABORATOIRE DE PARASITOLOGIE-MYCOLOGIE |
| DRACENIE-DRAGUIGNAN | DR | ERIC GARDIEN | CH DRACENIE-DRAGUIGNAN | LABORATOIRE DE PARASITOLOGIE-MYCOLOGIE |
| DUNKERQUE | DR | ANNICK VERHAEGHE | CH DUNKERQUE | LABORATOIRE DE PARASITOLOGIE-MYCOLOGIE |
| EPERNAY | DR | ELISABETH BUFFET | CH EPERNAY | LABORATOIRE DE PARASITOLOGIE-MYCOLOGIE |
| FALAISE | DR | G. GALLOU | CH FALAISE | LABORATOIRE DE PARASITOLOGIE-MYCOLOGIE |
| FOIX-VAL D'ARIEGE | DR | JEAN-BERNARD POUX | CH FOIX-VAL D'ARIEGE | LABORATOIRE DE PARASITOLOGIE-MYCOLOGIE |
| FONTAINEBLEAU | DR | CATHERINE BENOIT | CH FONTAINEBLEAU | LABORATOIRE DE PARASITOLOGIE-MYCOLOGIE |
| FREJUS-ST-RAPHAËL | DR | ANNIE MOTARD-PICHELOUP | CH FREJUS-ST RAPHAEL | LABORATOIRE DE BIOLOGIE MEDICALE |
| GONESSE | DR | CAROLE POUPON | CH GONESSE | LABORATOIRE DE PARASITOLOGIE-MYCOLOGIE |
| GRASSE | DR | N. DE POOTER | CH GRASSE | LABORATOIRE DE PARASITOLOGIE-MYCOLOGIE |
| GRENOBLE | DR | CECILE GARNAUD | CHU GRENOBLE | LABORATOIRE DE PARASITOLOGIE-MYCOLOGIE |
| GRENOBLE | DR | DANIELE MAUBON | CHU GRENOBLE | LABORATOIRE DE PARASITOLOGIE-MYCOLOGIE |
| GRENOBLE | DR | MARIE GLADYS ROBERT | CHU GRENOBLE | LABORATOIRE DE PARASITOLOGIE-MYCOLOGIE |
| LA REUNION | DR | CYNTHIA PIANETTI | CH LA REUNION | LABORATOIRE DE PARASITOLOGIE-MYCOLOGIE |
| LA ROCHELLE | DR | ANA MENDES-MOREIRA | CH LA ROCHELLE | LABORATOIRE D'HEMATOLOGIE |
| LA ROCHELLE | DR | SYLVAIN MERMOND | CH LA ROCHELLE | LABORATOIRE D'HEMATOLOGIE |
| LA ROCHE-SUR-YON | DR | GILBERT LORRE | CHD LA ROCHE-SUR-YON | LABORATOIRE DE BIOLOGIE MEDICALE |
| LA ROCHE-SUR-YON | DR | MARION LETERRIER | CHD LA ROCHE-SUR-YON | LABORATOIRE DE BIOLOGIE MEDICALE |
| LA ROCHE-SUR-YON | DR | THOMAS GUIMARD | CHD LA ROCHE-SUR-YON | SERVICE MEDECINE POST-URGENCE |
| LAGNY-SUR-MARNE | DR | VERONIQUE JAN-LASSERRE | CH LAGNY SUR MARNE | LABORATOIRE DE PARASITOLOGIE-MYCOLOGIE |
| LAVAL | DR | Axelle PAQUIN | CH LAVAL | LABORATOIRE DE BIOLOGIE MEDICALE |
| LAVAL | DR | Didier JAN | CH LAVAL | LABORATOIRE DE BIOLOGIE MEDICALE |
| LE CHESNAY | DR | FABRICE BRUNEEL | CH ANDRE MIGNOT | SERVICE DE REANIMATION MEDICO-CHIRURGICAL |
| LE HAVRE | DR | JEAN-PIERRE HURST | CH JACQUES MONOD | LABORATOIRE D'HEMATOLOGIE |
| LE HAVRE | DR | MURIEL SILVA | CH JACQUES MONOD | LABORATOIRE D'HEMATOLOGIE |
| LE HAVRE | DR | CELINE MALASSIGNE | CH LE HAVRE | LABORATOIRE DE PARASITOLOGIE-MYCOLOGIE |
| LE HAVRE | DR | SILVA | CH LE HAVRE | LABORATOIRE DE PARASITOLOGIE-MYCOLOGIE |
| LE MANS | DR | AURELIE BEAUDRON | CH LE MANS | LABORATOIRE DE BIOLOGIE MEDICALE |
| LE MANS | DR | BENJAMIN AUBRY | CH LE MANS | LABORATOIRE DE BIOLOGIE MEDICALE |
| LE MANS | DR | PASCALE PENN | CH LE MANS | LABORATOIRE DE BIOLOGIE MEDICALE |
| LILLE | DR | ANNE-SOPHIE DELEPLANCQUE | CHRU LILLE | INSTITUT DE MICROBIOLOGIE- CENTRE DE BIOLOGIE- PATHOLOGIE |
| LILLE | DR | BOUALEM SENDID | CHU LILLE | LABORATOIRE DE PARASITOLOGIE - MYCOLOGIE |
| LILLE | DR | EMILIE FREALLE | CHU LILLE | LABORATOIRE DE PARASITOLOGIE - MYCOLOGIE |
| LILLE | DR | LEROY JORDAN | CHU LILLE | LABORATOIRE DE PARASITOLOGIE - MYCOLOGIE |
| LIMOGES | DR | DANIEL AJZENBERG | CHU LIMOGES | LABORATOIRE DE PARASITOLOGIE-MYCOLOGIE |
| LIMOGES | PR | JEAN-FRANÇOIS FAUCHER | CHU LIMOGES | SERVICE DES MALADIES INFECTIEUSES |
| LIMOGES | PR | MARIE-LAURE DARDE | CHU LIMOGES | LABORATOIRE DE PARASITOLOGIE-MYCOLOGIE |
| LONGJUMEAU | DR | PATRICIA BARBUT | GH LONGJUMEAU | LABORATOIRE DE BIOLOGIE MEDICALE |
| LYON | PR | STEPHANE PICOT | CHU LYON CROIX ROUSSE | LABORATOIRE DE PARASITOLOGIE-MYCOLOGIE |
| MANTES-LA-JOLIE | DR | MARIE-LAURE BIGEL | CH MANTES-LA-JOLIE | LABORATOIRE DE PARASITOLOGIE-MYCOLOGIE |
| MARSEILLE | DR | BRUNO PRADINE | CERBA/IMTSSA | LABORATOIRE DE PARASITOLOGIE-MYCOLOGIE |
| MARSEILLE | DR | MARYLIN MADAMET | CERBA/IMTSSA | LABORATOIRE DE PARASITOLOGIE-MYCOLOGIE |
| MARSEILLE | DR | CORALIE L'OLLIVIER | CH TIMONE, IHU | LABORATOIRE DE PARASITOLOGIE-MYCOLOGIE |
| MARSEILLE | DR | STEPHANE RANQUE | CH TIMONE, IHU | LABORATOIRE DE PARASITOLOGIE-MYCOLOGIE |
| MARSEILLE | DR | PHILIPPE MINODIER | CHU AP-HM NORD | SERVICE DE NEONATALOGIE |
| MARSEILLE | DR | PHILIPPE PAROLA | CHU AP-HM NORD | SERVICE DE PATHOLOGIE INFECTIEUSE ET TROPICALE |
| MARSEILLE | PR | FABRICE SIMON | HIA MARSEILLE LAVERAN | SERVICE DE PATHOLOGIE INFECTIEUSE ET TROPICALE |
| MARSEILLE | DR | SAVINI HELENE | HIA MARSEILLE LAVERAN | SERVICE DE PATHOLOGIE INFECTIEUSE ET TROPICALE |
| MARTINIQUE | DR | DESBOIS-NOGARD | CHU DE LA MARTINIQUE | LABORATOIRE DE PARASITOLOGIE-MYCOLOGIE |
| MAYOTTE | DR | JF LEPERE | CH MAYOTTE | CMR DZOUMOGNE |
| MEAUX | DR | HELENE BROUTIER | CH MEAUX | LABORATOIRE D'HEMATOLOGIE |
| MONTAUBAN | DR | F. FEVRIER | CH MONTAUBAN | LABORATOIRE DE PARASITOLOGIE-MYCOLOGIE |
| MONTPELLIER | DR | NATHALIE BOURGEOIS | CHU MONTPELLIER | LABORATOIRE DE PARASITOLOGIE-MYCOLOGIE |
| MULHOUSE | DR | CAROLINE LOHMANN | GHR MULHOUSE | LABORATOIRE DE BIOLOGIE MEDICALE |
| MULHOUSE | DR | JEAN-MARIE DELARBRE | GHR MULHOUSE | LABORATOIRE DE BIOLOGIE MEDICALE |
| MULHOUSE | DR | ALAIN GRAVET | GHR MULHOUSE | LABORATOIRE DE BIOLOGIE MEDICALE |
| MULHOUSE | DR | FRANÇOISE SCHMITT | GHR MULHOUSE | LABORATOIRE DE BIOLOGIE MEDICALE |
| NANCY | DR | ANNE DEBOURGOGNE | CHU NANCY | LABORATOIRE DE PARASITOLOGIE-MYCOLOGIE |
| NANCY | DR | MARIE-CLAIRE MACHOUART | CHU NANCY | LABORATOIRE DE BACTERIOLOGIE |
| NANTES | DR | FAKHRI JEDDI | CHU NANTES | LABORATOIRE DE PARASITOLOGIE - MYCOLOGIE |
| NANTES | DR | ROSE-ANNE LAVERGNE | CHU NANTES | LABORATOIRE DE PARASITOLOGIE - MYCOLOGIE |
| NICE | DR | CHRISTELLE POMARES ESTRAN | CHU NICE | LABORATOIRE DE PARASITOLOGIE-MYCOLOGIE |
| NICE | DR | PASCAL DELAUNAY | CHU NICE | LABORATOIRE DE PARASITOLOGIE-MYCOLOGIE |
| NICE | PR | PIERRE MARTY | CHU NICE | LABORATOIRE DE PARASITOLOGIE-MYCOLOGIE |
| NIMES | DR | MILENE SASSO | CHU CAREMEAU | LABORATOIRE DE BIOLOGIE MEDICALE |
| NIMES | DR | VICTOR MERCIER | CHU CAREMEAU | LABORATOIRE DE BIOLOGIE MEDICALE |
| NIORT | DR | ALI CHERIF TOUIL | CH NIORT | LABORATOIRE DE BIOLOGIE MEDICALE |
| NIORT | DR | BERNADETTE BURET | CH NIORT | LABORATOIRE DE BIOLOGIE MEDICALE |
| ORLEANS | DR | AURELIE GUIGON | CHU ORLEANS | LABORATOIRE DE PARASITOLOGIE-MYCOLOGIE |
| ORLEANS | DR | DIDIER POISSON | CHU ORLEANS | LABORATOIRE DE PARASITOLOGIE-MYCOLOGIE |
| ORLEANS | DR | JEROME GUINARD | CHU ORLEANS | LABORATOIRE DE PARASITOLOGIE-MYCOLOGIE |
| PARIS | DR | AGNES DURAND | INSTITUT PASTEUR PARIS | LABORATOIRE VOLONTAIRES |
| PARIS | DR | HELENE LAPILLONNE | CH ARMAND TROUSSEAU | LABORATOIRE D'HEMATOLOGIE |
| PARIS | DR | NATHALIE DESUREMAIN | CH ARMAND TROUSSEAU | SERVICE DE PEDIATRIE GENERALE ET AVAL DES URGENCES |
| PARIS | DR | EMILIE SITTERLE | CH NECKER ENFANTS MALADES | SERVICE DE MICROBIOLOGIE CLINIQUE |
| PARIS | DR | FREDERIC SORGE | CH NECKER ENFANTS MALADES | DEPARTEMENT DE PEDIATRIE |
| PARIS | DR | MARIE-ELISABETH BOUGNOUX | CH NECKER ENFANTS MALADES | SERVICE DE MICROBIOLOGIE CLINIQUE |
| PARIS | DR | NADIA GUENNOUNI | CH NECKER ENFANTS MALADES | SERVICE DE MICROBIOLOGIE CLINIQUE |
| PARIS | DR | ALEXANDRA TIELLI | CH ROBERT DEBRE | SERVICE DE MALADIES INFECTIEUSES |
| PARIS | DR | ODILE FENNETEAU | CH ROBERT DEBRE | SERVICE DE MALADIES INFECTIEUSES |
| PARIS | PR | PR ALBERT FAYE | CH ROBERT DEBRE | SERVICE DE MALADIES INFECTIEUSES |
| PARIS | DR | YAYE SENGHOR | CH SAINT JOSEPH | SERVICE DE MICROBIOLOGIE |
| PARIS | DR | ALICIA MORENO-SABATIER | CH SAINT-ANTOINE | LABORATOIRE DE PARASITOLOGIE-MYCOLOGIE |
| PARIS | DR | SAMIA HAMANE | CH SAINT-LOUIS | LABORATOIRE DE PARASITOLOGIE-MYCOLOGIE |
| PARIS | DR | STEPHANE BRETAGNE | CH SAINT-LOUIS | LABORATOIRE DE PARASITOLOGIE-MYCOLOGIE |
| PARIS | DR | GHANIA BELKACEM BELKADI | CH TENON | LABORATOIRE DE PARASITOLOGIE-MYCOLOGIE |
| PARIS | DR | PIERRE MORNAND | CH TROUSSEAU | LABORATOIRE DE PARASITOLOGIE-MYCOLOGIE |
| PARIS | DR | CLAIRE AUGE | CHU BICHAT-CLAUDE BERNARD | LABORATOIRE DE PARASITOLOGIE-MYCOLOGIE |
| PARIS | PR | ENRIQUE CASALINO | CHU BICHAT-CLAUDE BERNARD | SERVICE DES URGENCES |
| PARIS | DR | GUILLAUME ESCRIOU | CHU BICHAT-CLAUDE BERNARD | CNR PALUDISME |
| PARIS | DR | JEROME CLAIN | CHU BICHAT-CLAUDE BERNARD | LABORATOIRE DE PARASITOLOGIE-MYCOLOGIE |
| PARIS | DR | LILIANE CICERON | CHU BICHAT-CLAUDE BERNARD | CNR PALUDISME |
| PARIS | MME | SARRASIN-HUBERT VERONIQUE | CHU BICHAT-CLAUDE BERNARD | CNR PALUDISME |
| PARIS | MME | MONIQUE LEMOINE | CHU BICHAT-CLAUDE BERNARD | CNR PALUDISME |
| PARIS | DR | NICOLAS ARGY | CHU BICHAT-CLAUDE BERNARD | LABORATOIRE DE PARASITOLOGIE-MYCOLOGIE |
| PARIS | PR | SANDRINE HOUZE | CHU BICHAT-CLAUDE BERNARD | LABORATOIRE DE PARASITOLOGIE-MYCOLOGIE |
| PARIS | DR | SANDRINE COJEAN | CHU BICHAT-CLAUDE BERNARD | CNR PALUDISME |
| PARIS | PR | SOPHIE MATHERON | CHU BICHAT-CLAUDE BERNARD | SERVICE DES MALADIES INFECTIEUSES |
| PARIS | DR | SYLVIE LARIVEN | CHU BICHAT-CLAUDE BERNARD | SERVICE DES MALADIES INFECTIEUSES |
| PARIS | DR | FREDERIC ARIEY | CHU COCHIN | LABORATOIRE DE PARASITOLOGIE-MYCOLOGIE |
| PARIS | DR | HELENE YERA | CHU COCHIN | LABORATOIRE DE PARASITOLOGIE-MYCOLOGIE |
| PARIS | DR | NAIMA DAHANE | CHU COCHIN | LABORATOIRE DE PARASITOLOGIE-MYCOLOGIE |
| PARIS | DR | ERIC DANNAOUI | CHU HEGP | UNITE DE PARASITOLOGIE - MYCOLOGIE. LABORATOIRE DE MICROBIOLOGIE |
| PARIS | DR | BENJAMIN  WYPLOSZ | CHU KREMLIN-BICETRE | SERVICE DES MALADIES INFECTIEUSES ET TROPICALES |
| PARIS | DR | ADELA ENACHE ANGOULVANT | CHU KREMLIN-BICETRE | LABORATOIRE DE PARASITOLOGIE-MYCOLOGIE |
| PARIS | DR | NADIA GUENNOUNI | CHU KREMLIN-BICETRE | LABORATOIRE DE PARASITOLOGIE-MYCOLOGIE |
| PARIS | PR | STEPHANE JAUREGUIBERRY | CHU KREMLIN-BICETRE | SERVICE DES MALADIES INFECTIEUSES ET TROPICALES |
| PARIS | DR | AMANDA LOPES | CHU LARIBOISIERE | MEDECINE INTERNE |
| PARIS | DR | ANNE-LISE MUNIER | CHU LARIBOISIERE | MEDECINE INFECTIEUSE |
| PARIS | DR | ANTHONY CHAUVIN | CHU LARIBOISIERE | SERVICE ACCUEIL DES URGENCES |
| PARIS | PR | BRUNO MEGARBANE | CHU LARIBOISIERE | REANIMATION MEDICALE ET TOXICOLOGIQUE |
| PARIS | DR | CAROLINE APARICIO | CHU LARIBOISIERE | CONSULTATION - POLYCLINIQUE |
| PARIS | DR | VERONIQUE DELCEY | CHU LARIBOISIERE | MEDECINE INFECTIEUSE |
| PARIS | DR | SAMIA HAMANE | CHU LARIBOISIERE/SAINT-LOUIS | LABORATOIRE DE PARASITOLOGIE |
| PARIS | DR | ALICE PERIGNON | CHU PITIE-SALPETRIERE | SERVICE DES MALADIES INFECTIEUSES |
| PARIS | MR | BILIGUI SYLVESTRE | CHU PITIE-SALPETRIERE | CNR PALUDISME |
| PARIS | PR | ERIC CAUMES | CHU PITIE-SALPETRIERE | SERVICE DES MALADIES INFECTIEUSES ET TROPICALES |
| PARIS | DR | ERIC KENDJO | CHU PITIE-SALPETRIERE | LABORATOIRE DE PARASITOLOGIE-MYCOLOGIE |
| PARIS | DR | GAY FREDERICK | CHU PITIE-SALPETRIERE | LABORATOIRE DE PARASITOLOGIE-MYCOLOGIE |
| PARIS | DR | ILHAME TANTAOUI | CHU PITIE-SALPETRIERE | LABORATOIRE DE PARASITOLOGIE-MYCOLOGIE |
| PARIS | DR | MARC THELLIER | CHU PITIE-SALPETRIERE | LABORATOIRE DE PARASITOLOGIE-MYCOLOGIE |
| PARIS | PR | RENAUD PIARROUX | CHU PITIE-SALPETRIERE | LABORATOIRE DE PARASITOLOGIE-MYCOLOGIE |
| PARIS | PR | MARTIN DANIS | CHU PITIE-SALPETRIERE | CNR PALUDISME |
| PARIS | PR | MAZIER DOMINIQUE | CHU PITIE-SALPETRIERE | LABORATOIRE DE PARASITOLOGIE-MYCOLOGIE |
| PARIS | PR | ALBERT FAYE | CHU ROBERT DEBRE | SERVICE DE PEDIATRIE GENERALE |
| PARIS | DR | JEAN-YVES SIRIEZ | CHU ROBERT DEBRE | SERVICE DES URGENCES |
| PARIS | DR | JEROME NAUDIN | CHU ROBERT DEBRE | SERVICE DE REANIMATION PEDIATRIQUE |
| PARIS | DR | LAUREN PULL | CHU ROBERT DEBRE | SERVICE DES URGENCES |
| PARIS | DR | ISABELLE TAWA | CMETE | LABORATOIRE DE BIOLOGIE MEDICALE |
| PARIS | DR | ALIOUNE NDOUR | INTS/INSERM | INSTITUT NATIONAL DE LA TRANSFUSION SANGUINE/ INSERM U1134 |
| PARIS | DR | BENOIT HENRI | INTS/INSERM | INSTITUT NATIONAL DE LA TRANSFUSION SANGUINE/ INSERM U1134 |
| PARIS | DR | CAMILLE ROUSSEL | INTS/INSERM | INSTITUT NATIONAL DE LA TRANSFUSION SANGUINE/ INSERM U1134 |
| PARIS | DR | CHARLOTTE CHAMBRION | INTS/INSERM | INSTITUT NATIONAL DE LA TRANSFUSION SANGUINE/ INSERM U1134 |
| PARIS | PR | PIERRE BUFFET | INTS/INSERM | INSTITUT NATIONAL DE LA TRANSFUSION SANGUINE/ INSERM U1134 |
| PARIS | DR | HAROLD NOËL | SPF | DEPARTEMENT MALADIES INFECTIEUSES |
| POISSY | DR | ROGER NABIAS | CHIPS POISSY/SAINT-GERMAIN-EN-LAYE | LABORATOIRE DE PARASITOLOGIE-MYCOLOGIE |
| POISSY | DR | JEAN YVES PELTIER | CHIPS POISSY/SAINT-GERMAIN-EN-LAYE | LABORATOIRE DE PARASITOLOGIE-MYCOLOGIE |
| POITIERS | DR | ANTOINE ELSENDOORN | CH POITIERS | LABORATOIRE DE PARASITOLOGIE-MYCOLOGIE |
| POITIERS | DR | ESTELLE PERRAUD-CATEAU | CH POITIERS | LABORATOIRE DE PARASITOLOGIE-MYCOLOGIE |
| POITIERS | DR | GWENAËL LE MOAL | CH POITIERS | SERVICE DE MALADIES INFECTIEUSES |
| PONTOISE | DR | MICHEL THIBAULT | CH PONTOISE | LABORATOIRE DE PARASITOLOGIE-MYCOLOGIE |
| PROVINS | DR | CHRISTIAN DURAND | CH PROVINS | LABORATOIRE DE PARASITOLOGIE-MYCOLOGIE |
| REIMS | DR | ANTOINE HUGUENIN | CHRU REIMS | LABORATOIRE DE PARASITOLOGIE-MYCOLOGIE |
| REIMS | DR | CATHY CHEMLA | CHRU REIMS | SERVICE DE MALADIES INFECTIEUSES |
| REIMS | DR | DOMINIQUE TOUBAS | CHRU REIMS | LABORATOIRE DE PARASITOLOGIE-MYCOLOGIE |
| REIMS | DR | FIROUZE BANISADR | CHRU REIMS | SERVICE DE MALADIES INFECTIEUSES |
| REIMS | DR | FREDERIQUE FOUDRINIER | CHRU REIMS | SERVICE DE MALADIES INFECTIEUSES |
| REIMS | DR | HUGUENIN | CHRU REIMS | SERVICE DE MALADIES INFECTIEUSES |
| RENNES | DR | MATTHIEU REVEST | CHU PONTCHAILLOU | SERVICE DES MALADIES INFECTIEUSES ET TROPICALES |
| RENNES | DR | SORYA BELAZ | CHU PONTCHAILLOU | LABORATOIRE DE PARASITOLOGIE-MYCOLOGIE |
| ROANNE | DR | JEAN-BENJAMIN MURAT | CH ROANNE | LABORATOIRE DE PARASITOLOGIE-MYCOLOGIE |
| ROUEN | DR | GILLES GARGALA | CHU CH.NICOLLE | LABORATOIRE DE PARASITOLOGIE - MYCOLOGIE |
| ROUEN | PR | LOÏC FAVENNEC | CHU CH.NICOLLE | LABORATOIRE DE PARASITOLOGIE - MYCOLOGIE |
| ROUEN | DR | MARION DEHAIS | CHU ROUEN | LABORATOIRE DE PARASITOLOGIE-MYCOLOGIE |
| SAINT-DENIS | DR | CELINE TOURNUS | CH SAINT-DENIS | LABORATOIRE DE PARASITOLOGIE-MYCOLOGIE |
| SAINT-ETIENNE | DR | PIERRE FLORI | CHU SAINT-ETIENNE | LABORATOIRE DE PARASITOLOGIE-MYCOLOGIE |
| SAINT-MALO | DR | ST. MALO-BIOL | CH SAINT-MALO | LABORATOIRE DE PARASITOLOGIE-MYCOLOGIE |
| SAINT-MANDE | PR | CECILE FICKO | HIA BEGIN | SERVICE DE PATHOLOGIE INFECTIEUSE ET TROPICALE |
| SAINT-MANDE | DR | SEBASTIEN LARRECHE | HIA BEGIN | LABORATOIRE DE BIOLOGIE MEDICALE |
| SAINT-NAZAIRE | DR | BOUDEN | CH SAINT-NAZAIRE | LABORATOIRE DE PARASITOLOGIE-MYCOLOGIE |
| STRASBOURG | DR | ABOU-BACAR AHMED | CHU STRASBOURG | LABORATOIRE DE PARASITOLOGIE-MYCOLOGIE |
| STRASBOURG | DR | ALEXANDER PFAFF | CHU STRASBOURG | LABORATOIRE DE PARASITOLOGIE-MYCOLOGIE |
| STRASBOURG | DR | DENIS FILISETTI | CHU STRASBOURG | LABORATOIRE DE PARASITOLOGIE-MYCOLOGIE |
| STRASBOURG | DR | ERMANNO CANDOLFI | CHU STRASBOURG | LABORATOIRE DE PARASITOLOGIE-MYCOLOGIE |
| STRASBOURG | DR | JULIE BRUNET | CHU STRASBOURG | LABORATOIRE DE PARASITOLOGIE-MYCOLOGIE |
| TOULON | DR | DAVID DELARBRE | HIA SAINTE-ANNE | LABORATOIRE DE BIOLOGIE MEDICALE |
| TOULON | DR | GUILLAUME MENARD | HIA SAINTE-ANNE | LABORATOIRE DE BIOLOGIE MEDICALE |
| TOULOUSE | DR | ANTOINE BERRY | CHU TOULOUSE-RANGUEIL | SERVICE DE PARASITOLOGIE-MYCOLOGIE |
| TOULOUSE | DR | PAMELA CHAUVIN | CHU TOULOUSE-RANGUEIL | SERVICE DE PARASITOLOGIE-MYCOLOGIE |
| TOURCOING | DR | GABRIELLE ROOSEN | CH GUY CHATILIEZ | LABORATOIRE DE PARASITOLOGIE-MYCOLOGIE |
| TOURCOING | DR | NICOLAS BLONDIAUX | CH GUY CHATILIEZ | LABORATOIRE DE PARASITOLOGIE-MYCOLOGIE |
| TOURCOING | DR | PIERRE PATOZ | CH GUY CHATILIEZ | LABORATOIRE DE PARASITOLOGIE-MYCOLOGIE |
| TOURS | DR | ADELAIDE CHESNAY | CHRU BRETONNEAU | LABORATOIRE DE PARASITOLOGIE-MYCOLOGIE |
| TOURS | PR | GUILLAUME DESOUBEAUX | CHRU BRETONNEAU | LABORATOIRE DE PARASITOLOGIE-MYCOLOGIE |
| TROYES | DR | FARIDA BENAOUDIA | CH TROYES | LABORATOIRE DE BIOLOGIE MEDICALE |
| TROYES | DR | MAXIME THOUVENIN | CH TROYES | LABORATOIRE DE BIOLOGIE MEDICALE |
| TROYES | DR | MOREAU-BENAOUDIA | CH TROYES | LABORATOIRE DE BIOLOGIE MEDICALE |
| VALENCIENNES | DR | MAZARS EDITH | CH VALENCIENNES | LABORATOIRE DE BIOLOGIE MEDICALE |
| VANNES | DR | ANNE CADY | CH BRETAGNE ATLANTIQUE | LABORATOIRE D'HEMATOLOGIE-MICROBIOLOGIE |
| VANNES | DR | GREGORY CORVAISIER | CH BRETAGNE ATLANTIQUE | LABORATOIRE D'HEMATOLOGIE-MICROBIOLOGIE |
| VANNES | DR | MARIE GOUSSEFF | CH BRETAGNE ATLANTIQUE | LABORATOIRE D'HEMATOLOGIE-MICROBIOLOGIE |
| VANNES | DR | PASCAL POUEDRAS | CH BRETAGNE ATLANTIQUE | LABORATOIRE D'HEMATOLOGIE-MICROBIOLOGIE |
| VANNES | DR | YOANN CRABOL | CH BRETAGNE ATLANTIQUE | LABORATOIRE D'HEMATOLOGIE-MICROBIOLOGIE |
| VERNON | DR | DIABY MOHAMED | CH VERNON | LABORATOIRE DE PARASITOLOGIE-MYCOLOGIE |
| VERSAILLES | DR | ODILE ELOY | CH ANDRE MIGNOT | LABORATOIRE DE BIOLOGIE MEDICALE |
| VILLENEUVE SAINT-GEORGES | DR | ALICE RAFFETIN | CHI VILLENEUVE SAINT-GEORGES | SERVICE DE MALADIES INFECTIEUSES |
| VILLENEUVE SAINT-GEORGES | DR | PAULINE CARAUX-PAZ | CHI VILLENEUVE SAINT-GEORGES | SERVICE DE MALADIES INFECTIEUSES |
| VILLENEUVE SAINT-GEORGES | DR | SYLVIE MAURELLET EVRARD | CHI VILLENEUVE SAINT-GEORGES | LABORATOIRE DE PARASITOLOGIE-MYCOLOGIE |
